# Phage receptor specificity drives cross-resistance patterns and governs fitness trade-offs during sequential resistance acquisition in *Salmonella*

**DOI:** 10.1093/ismejo/wrag077

**Published:** 2026-04-11

**Authors:** Yingting Wu, Jing Yu, Weilai Tao, Jie Wu, Yumeng Gan, Yuxuan Wang, Xin Zhao, Xiaojing Hao, Qian Zhang, Hongning Wang, Anyun Zhang

**Affiliations:** Animal Disease Prevention and Green Development Key Laboratory of Sichuan Province, College of Life Sciences, Sichuan University, Chengdu, Sichuan 610065, China; Animal Disease Prevention and Green Development Key Laboratory of Sichuan Province, College of Life Sciences, Sichuan University, Chengdu, Sichuan 610065, China; Animal Disease Prevention and Green Development Key Laboratory of Sichuan Province, College of Life Sciences, Sichuan University, Chengdu, Sichuan 610065, China; Animal Disease Prevention and Green Development Key Laboratory of Sichuan Province, College of Life Sciences, Sichuan University, Chengdu, Sichuan 610065, China; Animal Disease Prevention and Green Development Key Laboratory of Sichuan Province, College of Life Sciences, Sichuan University, Chengdu, Sichuan 610065, China; Animal Disease Prevention and Green Development Key Laboratory of Sichuan Province, College of Life Sciences, Sichuan University, Chengdu, Sichuan 610065, China; Animal Disease Prevention and Green Development Key Laboratory of Sichuan Province, College of Life Sciences, Sichuan University, Chengdu, Sichuan 610065, China; Qingdao Animal Husbandry Workstation, Qingdao 266100, China; Qingdao Animal Husbandry Workstation, Qingdao 266100, China; Animal Disease Prevention and Green Development Key Laboratory of Sichuan Province, College of Life Sciences, Sichuan University, Chengdu, Sichuan 610065, China; Animal Disease Prevention and Green Development Key Laboratory of Sichuan Province, College of Life Sciences, Sichuan University, Chengdu, Sichuan 610065, China

**Keywords:** phage receptors, phage resistance, co-evolution, evolutionary trade-offs

## Abstract

Phages infect bacteria by binding to specific surface receptors, driving co-evolution in microbial communities and offering therapeutic potential. However, how receptor specificity shapes the cross-resistance patterns and evolutionary trade-offs during phage-bacteria co-evolution remains unclear. Here, we investigated the genetic basis and fitness trade-offs of phage resistance in *Salmonella* to phages targeting O-antigen, core oligosaccharide, and BtuB (TonB-dependent receptor for vitamin B_12_) under individual or combinatorial pressures. The interaction matrices between phage-resistant strains and phages targeting three different receptors showed that bacterial cross-resistance to phages depends on the receptor type. Lipopolysaccharide (LPS) truncation conferred cross-resistance to phages targeting either the O-antigen or core oligosaccharide; whereas resistance to phages targeting BtuB occurred exclusively through mutations in the *btuB* gene. For LPS receptors whose biosynthesis involves multiple genes, the fitness cost associated with phage resistance is gene-specific. Among mutations conferring resistance to both O-antigen-targeting and core-targeting phages, those in the *rfaJ* gene exhibited the lowest fitness cost. The three-phage combination targeting three receptors exhibited potent antibacterial effects. Under this selective pressure, *Salmonella* developed resistance through receptor modification. Resistance to O-antigen-targeting and core-targeting phages emerged first through mutations in LPS biosynthesis genes, with mutations in the *rfaJ* gene dominating. Subsequently, mutations in the *btuB* gene accumulated to resist BtuB-targeting phages, ultimately evading predation by all three phages. Our results reveal receptor-driven evolutionary trade-offs and sequential resistance acquisition in *Salmonella* under multiple phages pressure, enhancing understanding of microbial interactions and informing phage therapy strategies.

## Introduction

Phages that infect bacteria are the most abundant and diverse biological entities on earth [1]. The long-standing co-evolution between bacteria and phages is an important driver for maintaining genetic diversity and shaping microbial communities [2]. In response to phage predation pressure, bacteria have evolved defense strategies targeting each stage of the phage life cycle, including surface receptor modification to block adsorption, restriction-modification and CRISPR systems to disrupt DNA replication and transcription, and abortive infection systems to prevent assembly and release [3]. Among these, receptor modification emerges as the most rapid and prevalent resistance strategy, directly destroying the initial interaction between phage and host bacteria [4–6]. In the natural environment, phages can target various structures on the bacterial surface, including lipopolysaccharide (LPS), extracellular polymers, flagella, and various outer membrane proteins (OMP) [7, 8]. These structures serve multiple functions in the host bacteria, and resistance to phages through receptor modification often comes at the cost of functions [9]. These fitness costs associated with phage resistance, such as reduced growth [10], attenuated virulence [1], and increased antibiotic sensitivity [12, 13], exert selection pressure that leads to evolutionary trade-offs in bacteria. The fitness costs incurred by surface receptor modifications are crucial factors in the evolutionary trade-offs for bacteria within microbial communities [14]. Although the evolution of bacterial resistance to an individual phage has been extensively studied, our understanding of how bacteria evolve trade-offs under pressures from diverse phages targeting different receptors remains limited. Studying complex interactions between bacteria and multiple phages can help reveal the evolutionary dynamics of bacterial genetics in microbial communities.

The widespread use of antibiotics has led to the emergence and spread of antibiotic-resistant bacteria, making the antibiotic resistance crisis one of the most significant global public health challenges [15]. *Salmonella* is an important zoonotic pathogen exhibiting high resistance rate to multiple antibiotics. It can be transmitted through contaminated food, causing food poisoning and potentially leading to systemic infections [16, 17]. Each year, ~115 million people are infected with *Salmonella*, resulting in ~370 000 deaths [18]. In response to the antibiotic resistance crisis, phage therapy is being revived and gradually applied in clinical medicine, animal husbandry, and food production [19, 20]. Compared to individual phages, a cocktail of multiple phages can enhance antibacterial efficacy by either increasing the fitness cost associated with phage resistance or delaying its evolution [21, 22]. The ideal phage cocktail consists of phages that target different receptors [23, 24]. Current phage cocktail research has moved from simple combinations to characterizing phage genomes and targeted receptors for optimal antibacterial design [25, 26], yet comprehensive guidelines remain lacking. Characterizing the extent and determinants of cross-resistance to other phages in phage-resistant strains, which evolved under specific phage selection pressure, will facilitate the rapid selection of effective phage combinations from established libraries. Furthermore, understanding the sequence and pattern of bacterial resistance evolution to phage cocktails can improve and optimize phage therapy.

To investigate the cross-resistance development dynamics and fitness trade-offs in bacteria under diverse phage pressures, this study utilized *Salmonella enterica* ATCC 13076 and a panel of eight virulent phages targeting three different receptors: the core oligosaccharide and O-antigen of LPS, and the OMP BtuB. We systematically characterized the cross-resistance patterns and genetic basis of *Salmonella* against these phages, quantified the fitness costs associated with phage-resistant genetic variants, and tracked the dynamics of resistance evolution over time under combinatorial phage pressure using whole-population genome sequencing. Our findings reveal that phage receptor specificity defines the set of genes that can be mutated to confer resistance, thereby shaping cross-resistance patterns and ultimately driving sequential resistance acquisition under combinatorial phage pressure through trade-offs in fitness costs. These results provide fundamental insights into the evolutionary dynamics of bacterial populations within natural microbial communities and establish a foundation for the rational design of phage cocktails.

## Materials and methods

### Bacterial strains and growth condition

*S. enterica* ATCC 13076 was used for phage isolation, construction of gene deletion strains, isolation of phage-resistant strains, determination of growth curves under phage selection pressure, and serial passaging culture with phage. Eighteen gene deletion strains were constructed using the pEcCas/pEcgRNA system based on CRISPR-Cas-assisted gene editing [27], and the primers used are shown in Table S1. Among these, 14 strains (Δ*rfaP*, Δ*rfaC*, Δ*rfaY*, Δ*rfaF*, Δ*rfaQ*, Δ*rfaG*, Δ*rfaB*, Δ*rfaI*, Δ*rfaJ*, Δ*rfaK*, Δ*rfbP*, Δ*OmpC*, Δ*btuB*, Δ*fliC*) were employed to identify phage receptors, whereas the remaining four (Δ*selB*, Δ*pabC*, Δ*WcaD*, Δ*tktA*) were used to evaluate the contribution of non-receptor genes to the phage resistance phenotype. All *Salmonella* strains were cultured in Lysogeny broth (LB) medium at 37°C.

### Isolation and purification of phage

Phages PhSAL1 to PhSAL8 were isolated from sewage and chicken manure using *S. enterica* ATCC 13076 as the host bacteria. Briefly, after mixing the chicken manure sample with phosphate-buffered saline (PBS), both the processed chicken manure and the sewage sample were centrifuged at 8000 rpm for 2 min, and their supernatants were filtered through 0.22 μm filters. Then, 5 ml of the filtrate and 1 ml of the host bacteria in the exponential growth phase were mixed with 20 ml of LB medium and cultured at 37°C for 6 h. The mixture was centrifuged and filtered under the same conditions, and the phage in the filtrate were purified by double agar overlay method [28]. The purification steps were repeated at least three times.

### Phage genome analysis

Genomic DNA from phage lysates containing at least 10^9^ particles was extracted using the Phage DNA Isolation Kit (Norgen Biotek, Cat. 46 800) and whole-genome sequencing (WGS) was performed using NovaSeq PE150 (Illumina). The sequencing raw data were quality controlled using fastp v0.20.0 [29]. Clean reads were then assembled de novo using SPAdes v3.12.0 [30] to generate contigs. High-depth sequences were extracted and aligned via BLASTN against the NCBI non-redundant nucleotide database to identify viral genomic sequences from the assembled contigs. Collinearity analysis was performed with MUMmer v3.1 [31] to determine the relative positions of contigs and to fill gaps between them. Finally, the assembly was polished using Pilon v1.18 [32] to obtain the complete phage genome sequence.

The complete phage genomes were annotated using Prokka v1.14.6 [33], and genome alignments were visualized using Easyfig v2.2.5 [34]. VIRIDIC [35] was used to calculate intergenomic similarities among phages, and the results are shown in Table S2. For comparative genomic analysis with the phage isolated in this study, genomes of classic phages from different genera were obtained from GenBank. Phages were classified at the family and genus levels using taxMyPhage v0.3.6 [36] in accordance with International Committee on Taxonomy of Viruses guidelines. PhageScope (https://phagescope.deepomics.org/) [37] was used to predict the phage lifestyle. A phylogenetic tree based on major capsid proteins was constructed with MEGA 11.0 using the neighbor-joining algorithm. The major capsid protein of isolated *Salmonella* phages and its homologous proteins were found by using BLASTX to manually search against non-redundant protein database in NCBI. The genome of the eight isolated phages was deposited at GenBank under accession no. PV988430-PV988437.

### Phage morphology identification

About 20 μl of purified phage concentrate was dropped onto the copper grid and allowed to stand for 3–5 min, then negatively stained with 2% phosphotungstic acid (pH = 7) for 10 min. The morphology of phage was identified using a transmission electron microscopy (TEM, JEM-2100Plus).

### Efficiency of plating

The efficiency of plating (EOP) on the gene deletion strain relative to that on wild-type (WT) strain was measured to identify phage receptors or to evaluate the contribution of non-receptor genes to phage resistance phenotypes. About 100 μl of WT strain or its deletion strain culture (OD_600_ of 0.6–0.8) was mixed with an equal volume of serially diluted phage lysate. The mixture was incubated at 37°C for 15–20 min, then 2–3 ml of soft LB agar containing 0.6% agar (w/v) was added and poured onto a solid LB agar plate. After overnight incubation at 37°C, plaques on the plates were counted to calculate the EOP (phage titer of the gene deletion strain/phage titer of WT strain). The inability of a phage to form plaques on a strain lacking a specific gene indicates that the gene encodes the phage receptor. For non-receptor genes, the EOP value can reflect the degree of phage resistance conferred by the deletion of the gene.

### Phage adsorption assay

The phage adsorption rates on the WT strain and the gene deletion strains were compared to further verify the type of receptor targeted by the phages. Specifically, 400 μl of either the WT strain or a gene deletion strain (10^8^ CFU) was mixed with 200 μl of phage lysate (2 × 10^5^ particles) and incubated statically at 37°C for 15 min, using LB medium as a control. After centrifugation at 13 000 rpm for 5 min, the supernatant was filtered through a 0.22 μm filter. The number of unadsorbed free phages in the filtrate was then determined. The phage adsorption rate was calculated as (initial phage titer - free phage titer) / initial phage titer. All experiments were performed in triplicate.

### Isolation of phage-resistant strains selected by individual phage

*S. enterica* ATCC 13076 was infected with individual phages (PhSAL1 to PhSAL8) and the surviving strains were isolated. About 100 μl of bacterial culture (OD_600_ of 0.6–0.8) was mixed with an equal volume of phage lysate (containing 10^8^ particles) and plated on a solid LB agar plate, followed by incubation at 37°C for 24 h. To avoid pseudoreplication and ensure the isolation of independent mutants, only one single colony was randomly selected from each LB plate (one plate per experimental replicate), with each clone considered to be selected from an independent phage infection event [38, 39]. The experiment was repeated 20 times for each phage strain, except for phage PhSAL5 (replicated 40 times). The selected colonies were streaked onto XLT4 plates and subjected to at least three successive rounds of streaking to obtain a purified strain devoid of phage. The purified strain was inoculated into fresh LB liquid medium and cultured overnight at 37°C. The next day, 1% of the overnight culture was transferred to fresh LB liquid medium and continued to culture until the OD_600_ was 0.6–0.8, then mixed with glycerol (final concentration 25%) and frozen at −80°C.

### Quantification of phage resistance

The strength of resistance to phages was quantified by measuring the relative bacterial growth (RBG) in the presence and absence of phages, as described previously [40–44]. The resistance strength of all isolated strains to phages PhSAL1 to PhSAL8 was determined. Resistance quantitative assays were performed in 96-well microplates in LB medium. Bacteria and phages were mixed at a multiplicity of infection (MOI) of 10 (10^5^ bacterial cells and 10^6^ phage particles), and supplemented with LB medium to a final volume of 150 μl. At least three replicates were performed for each strain, with no phage added as a control. The absorbance was measured initially and after 8 h of incubation at 37°C to calculate RBG (equation 1), with a value of 1 indicating complete resistance (growth was equal in the presence and absence of phages) and a value of 0 indicating complete sensitivity (no growth in the presence of phage). For some phage-resistant strains with slow growth rates, the endpoint time was extended to 16 h. The RBG values of these strains are shown in Table S3. To define the thresholds for phage sensitivity and resistance, kernel density estimation was performed on the mean RBG values (180 strains × 8 phages). Two major peaks (0.034 and 0.977) were identified in the density curve using the findpeaks function in the R package *pracma* [45], and the valley between them was located, yielding an initial threshold of 0.511. The robustness of this estimate was assessed by bootstrap resampling (1000 iterations), which provided a 95% confidence interval. Based on this, the final thresholds were defined as follows: sensitive (RBG ≤ 0.49), intermediate (0.49 < RBG < 0.53), and resistant (RBG ≥ 0.53). The resulting classification is visualized using the R package *ggplot2* [46] (Fig. S3).

For phage *i*, bacteria *j*:


(1) \begin{equation*} {RBG}_{ij}=\frac{{\left[{Abs}_{600}\left(t=8\ h\right)-{Abs}_{600}\left(t=0\ h\right)\right]}_{ij}}{{\left[{Abs}_{600}\left(t=8\ h\right)-{Abs}_{600}\left(t=0\ h\right)\right]}_{controlj}} \end{equation*}

### Bacterial genome sequencing and mutation analysis

WGS was used to identify genetic mutations within individual bacterial strains. Genomic DNA was extracted from pelleted overnight bacterial cultures using the TIANamp Bacteria DNA Kit (TIANGEN, Cat No. DP302). Sequencing was performed on the NovaSeq 6000 System (Illumina), and quality control of the sequencing raw data was conducted using fastp v0.20.0 [29]. Next, single nucleotide polymorphisms (SNPs) and insertion/deletion (indel) variants within the quality-controlled fastq files were identified using the *breseq* pipeline v0.38.3 [47]. Only loss-of-function mutations, including nonsense mutations, missense mutations, frameshift mutations, and large indels, were retained. Furthermore, preexisting genetic background mutations detected in the WT strain were excluded from analysis, as they were not generated in response to phage pressure. Mutations in individual strains were called using the default parameters.

Whole-population genome sequencing was used to determine the frequency of genetic mutations across bacterial populations. Mixtures of phages and host bacteria collected at different time points during co-culture were subjected to genomic DNA extraction, sequencing, quality control, and mutation analysis using the same methods described above. To determine the mutation frequency within the bacterial population, the *breseq* pipeline v0.38.3 was employed with minor modifications: default parameters were applied in combination with the -p flag for polymorphism detection, and a minimum frequency threshold was set at 5%. The detailed genetic mutations and accession number of the isolated strains and bacterial populations are shown in Table S4.

### LPS extraction and silver staining

LPS was extracted from 100 ml of overnight bacterial culture using a Lipopolysaccharide Extraction Kit (BestBio, Cat No. BB-31302). The extracted LPS was freeze-dried and then dissolved in 100 μl distilled water. Next, add 5 μl of protein loading buffer (Beyotime, Cat No. P0015) to 20 μl of LPS and heat in boiling water for 5 min. Denatured LPS samples were separated by 12% sodium dodecyl sulfate polyacrylamide gel electrophoresis (SDS-PAGE) (ACE biotechnology, Cat No. ET12010Gel) and subsequently silver stained using the Lipopolysaccharide Rapid Silver Staining Kit (Zeye Biotechnology, Cat No. ZY2369a).

### Measurement of bacterial maximum growth rate

The maximum bacterial growth rate served as an indicator of fitness cost. The fitness costs associated with phage resistance were calculated by measuring the growth of mutant strains compared with the WT strain. About 100 μl of bacterial culture with a density of 10^6^ CFU/ml was inoculated into 96-well microplates (final volume of 150 μl), incubated at 37°C for 24 h and measured the absorbance every 30 min (λ = 600 nm). All experiments were performed in triplicate. The R package *gcplyr* [48] was used to evaluate the maximum growth rate of bacteria. The relative fitness cost of the mutant strain was the maximum growth rate of the mutant strain divided by the maximum growth rate of the ancestral strain.

### Bacterial competition assays

The competition assay between phage-resistant strains and WT strain was performed as previously described [49], with minor modifications. Briefly, overnight cultures of the phage-resistant strains and the wild-type strain were diluted 100-fold and plated separately for colony counting determine the initial density (T₀). The two strains were then mixed in equal volumes (100 μl each), and inoculated into 5 ml of fresh LB medium, followed by shaking incubation at 37°C for 24 h. After incubation, the culture was serially diluted and plated to determine the final total bacterial density. To distinguish between the phage-resistant and WT strains, 20 randomly selected single colonies were tested for phage sensitivity using the plaque assay; colonies that forming clear plaques were identified as WT strain. The final densities (T_24_) of the phage-resistant and WT strains at 24 h were calculated based on their respective proportions in the mixture. The relative competitive fitness (W) of the phage-resistant strain was then calculated (equation 2). All experiments were performed in triplicate.

For phage-resistant strain R and WT strain S:


(2) \begin{equation*} W=\frac{\mathit{\ln}\left[{T}_R\left(t=24\ h\right)/{T}_R\left(t=0\ h\right)\right]}{\mathit{\ln}\left[{T}_S\left(t=24\ h\right)/{T}_S\left(t=0\ h\right)\right]} \end{equation*}

### *Salmonella* transposon mutant library screening

As previously described with minor modifications, a *Mariner* transposon mutant library of *S. enterica* ATCC 13076 was constructed using plasmid pSAM_Ec and *E.coli* X7213 as the donor strain [50]. The mutant library was co-infected with an equal mixture of phages PhSAL1, PhSAL5, and PhSAL7 at a MOI of 0.01, with an uninfected mutant library serving as the control. All experiments were performed in triplicate. Following incubation at 37°C with shaking for 8 h, bacterial cells were pelleted by centrifugation for DNA extraction. The sequencing library was prepared as previously described [51] and then subjected to sequencing on the NovaSeq 6000 System (Illumina). The Tradis pipeline [52] was employed to identify mutant genes that exhibit significant enrichment following phage infection.

### Bacterial growth curves for phage inhibition assays

Growth curves were determined for *S. enterica* ATCC 13076 treated with: no phage, an individual phage, or combinations of phage targeting either same or different receptors. Phage combinations targeting the same receptor include: PhSAL1 and PhSAL2; PhSAL1, PhSAL2, and PhSAL3; PhSAL6 and PhSAL7; PhSAL6, PhSAL7, and PhSAL8. Phage combinations targeting different receptors include: PhSAL1 and PhSAL5; PhSAL1 and PhSAL7; PhSAL5 and PhSAL7; PhSAL1, PhSAL5 and PhSAL7. Bacterial suspensions and phages were inoculated into 96-well plates at a MOI of 1. The initial density was 10^5^ bacterial cells or phage particles per well, and supplemented with LB medium to a final volume of 200 μl. In combinations of multiple phages, the total amount of phages remained constant, with each phage added equally. Then the 96-well plates were incubated at 37°C for 30 h, and absorbance (λ = 600 nm) was measured at 0, 2, 4, 6, 8, 10, 12, 20, and 30 h. All experiments were performed in eight replicates. The area under the curve (AUC) was calculated using the R package *gcplyr* [48].

### Co-culture of phages and host bacteria

*S. enterica* ATCC 13076 was co-cultured for 7 days either with individual phages (PhSAL1, PhSAL5, and PhSAL7) or a three-phage combination (PhSAL1, PhSAL5 and PhSAL7; PhSAL2, PhSAL5, and PhSAL8). Bacterial cultures were initiated by inoculating ~10^5^ cells into 10 ml of LB medium. Phages were added at a MOI of 1; for the three-phage combination treatment, each phage was added in equal amounts to achieve a total MOI of 1 (10^5^ particles). All cultures were incubated at 37°C with shaking at 180 rpm. Each treatment condition was performed in triplicate, alongside control cultures without phage addition. Every 24 h, 100 μl of the mixed culture was transferred into 10 ml of fresh LB medium and 10 discrete colonies were isolated from three replicates of each treatment group. After purification through at least three successive streak passages, the phage susceptibility of these isolates was assessed by measuring the RBG. For the phage combination comprising PhSAL1, PhSAL5, and PhSAL7, genomic DNA was extracted from the mixture at 12 h and 1, 3, 5, and 7 day of co-culture for whole-population genome sequencing. Concurrently, phage-resistant strains isolated at 1, 3, 5, and 7 day were selected for WGS. For the phage combination comprising PhSAL2, PhSAL5, and PhSAL8, phage-resistant strains isolated at 1 and 7 days of co-culture were selected for WGS.

### Statistical analysis

All statistical analyses were performed using IBM SPSS Statistics v23.0. Data are presented as mean ± standard deviation (SD) or as median with interquartile range, as appropriate. Statistical significance was defined as a two-tailed *P* value ≤0.05 (^*^ indicates *P* ≤ 0.05, ^**^ indicates *P* ≤ 0.01). The test statistics are shown in Table S5. For comparisons of phage adsorption rates and efficiency of plating (EOP) between gene deletion strains and the WT strain, we used unpaired Student’s t-tests. For the maximum growth rate data, the non-parametric Mann–Whitney U test was used to assess the statistical significance of the changes in phage-resistant strains relative to WT strain. For bacterial competitive ability data, the one-sample t-test was used to assess the significance of the change in the competitive ability of phage-resistant strains relative to WT strains. For growth curve data, we performed analysis of variance (ANOVA) based on AUC followed by Dunnett's T3 test to assess significance across different phage treatment groups. Statistical details are also included in the respective figure legends. For the details on experimental replicates see the Methods section.

## Results

### Cross-resistance of bacteria to different phages is receptor-dependent

In this study, we first investigated whether the bacterial surface receptor used by the phage is a critical factor in causing cross-resistance of specific phage-resistant strains to other phages. We used *S. enterica* ATCC 13076 and eight newly isolated virulent phages (named PhSAL1-PhSAL8) as the research materials. The genome sizes of these phages ranged from 43.7 to 124.9 kb, and no deleterious genes were annotated (Table 1). Phylogenetic analysis assigned them to three distinct genera: *Felixounavirus* (PhSAL1-PhSAL4), *Jerseyvirus* (PhSAL5), and *Epseptimavirus* (PhSAL6-PhSAL8) (Fig. S1A). PhSAL1-PhSAL4 showed 90.2%–98.8% intergenomic similarity to each other and 86.6%–92.0% similarity to other known *Felixounavirus* phages. PhSAL5 showed 66.0%–81.9% intergenomic similarity with known *Jerseyvirus* phages. PhSAL6-PhSAL8 showed 80.0%–85.7% intergenomic similarity to each other and 79.4%–84.1% similarity to other known *Epseptimavirus* phages (Fig. S1B, Table S2). TEM observations revealed that all PhSAL1-PhSAL8 possess tails; specifically, PhSAL1-PhSAL4 have contractile tails, whereas PhSAL5-PhSAL8 have long, non-contractile tails (Fig. S1C).

**Table 1:** Genomic information of isolated phages.

Phage	Taxonomy (Family, Genus)	Genome size (bp)	ORF number
PhSAL1	*Andersonviridae*, *Felixounavirus*	84 489	133
PhSAL2	*Andersonviridae*, *Felixounavirus*	86 761	144
PhSAL3	*Andersonviridae*, *Felixounavirus*	85 919	141
PhSAL4	*Andersonviridae*, *Felixounavirus*	86 264	142
PhSAL5	*Sarkviridae*, *Jerseyvirus*	43 711	65
PhSAL6	*Demerecviridae*, *Epseptimavirus*	120 302	208
PhSAL7	*Demerecviridae*, *Epseptimavirus*	122 094	209
PhSAL8	*Demerecviridae*, *Epseptimavirus*	124 461	207

**Figure 1:**
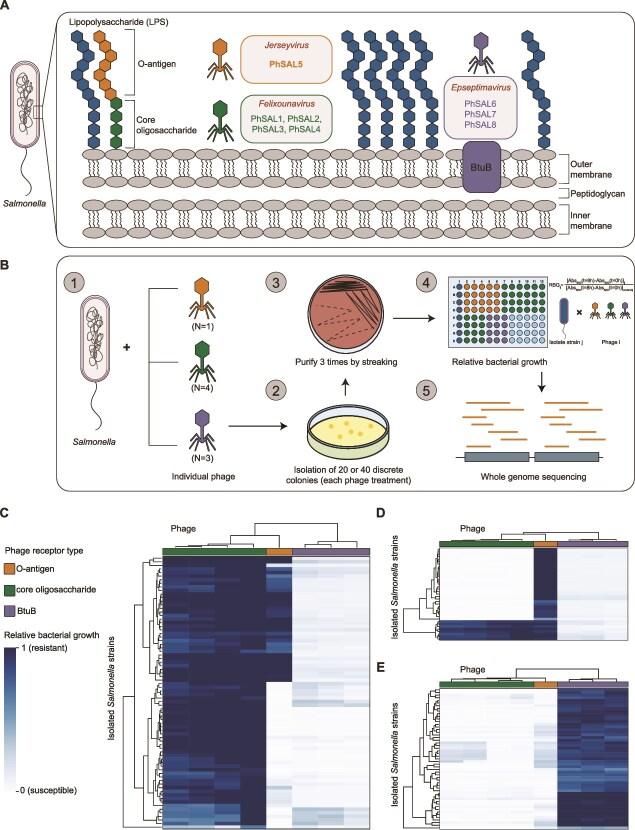
Characterization of cross-resistance patterns in *Salmonella* against eight virulent phages. (**A**) Bacterial surface structures targeted by eight phages. (**B**) Flowchart for isolation of phage-resistant strains and determination of cross-resistance. *Salmonella enterica* ATCC 13076 was individually challenged with each phage and plated on LB agar plates. Following 24 h incubation at 37°C, 20 or 40 colonies were randomly selected for each phage treatment and subjected to three streak purification cycles. Cross-resistance was quantified by measuring RBG in the presence and absence of each phage, followed by WGS to identify the genetic determinants of phage resistance. (**C–E**) Hierarchical clustered heatmap showing the cross-resistance profiles of isolated phage-resistant strains against different phages. Strains were isolated under pressure from phages targeting the core **C**, O-antigen (**D**), or BtuB (**E**). The vertical axis indicates phage-resistant strains, and the horizontal axis indicates phages.

To identify receptors essential for phage infection, we constructed gene deletion strains for common receptor types. A total of 14 strains were constructed, including 10 with deletions in genes involved in LPS core oligosaccharide biosynthesis (Δ*rfaP*, Δ*rfaC*, Δ*rfaY*, Δ*rfaF*, Δ*rfaQ*, Δ*rfaG*, Δ*rfaB*, Δ*rfaI*, Δ*rfaJ*, and Δ*rfaK*), one with a deletion in the gene required for LPS O-antigen biosynthesis (Δ*rfbP*), and three with deletions in genes encoding OMPs (Δ*ompC*, Δ*btuB*, and Δ*fliC*). These deletion strains were then used to determine the EOP of the eight phages relative to the WT strain. The results indicated that the core oligosaccharide structure of LPS was essential for plaque formation by phages PhSAL1-PhSAL5; whereas the O-antigen structure of LPS was essential for PhSAL5 only (Fig. S2A). This difference reveals their distinct receptor targets. Because LPS is composed of lipid A, the core oligosaccharide (hereafter referred to as the “core”), and the O-antigen, assembled from the inner to the outer region, the core is required for the attachment of the O-antigen (its absence leads to loss of both structures), but the O-antigen is not required for core integrity [53]. Therefore, the core is the candidate receptor for PhSAL1-PhSAL4, whereas the O-antigen is for PhSAL5. For phages PhSAL6-PhSAL8, deletion of *btuB* gene abolished plaque formation, suggesting that OMP BtuB serves as a candidate receptor (Fig. S2A). Further comparison of the adsorption rates of these phages before and after the deletion of candidate receptor genes indicated that core deletion significantly reduced adsorption of PhSAL1-PhSAL5; O-antigen deletion reduced adsorption of PhSAL5 only; whereas *btuB* deletion significantly reduced adsorption of PhSAL6-PhSAL8 (Fig. S2B). These results collectively identified the receptors targeted by each phage. Overall, we isolated eight phages targeting three different receptors: core (PhSAL1-PhSAL4), O-antigen (PhSAL5), and BtuB (PhSAL6-PhSAL8) (Fig. 1A).

To isolate phage-resistant strains, bacteria were infected with individual phages. We selected 20 resistant clones for each focal phage, except for PhSAL5 (only this one targeting the O-antigen), for which 40 resistant clones were selected. The strength of phage resistance was quantified by measuring the RBG of all isolates in the presence and absence of each phage (Table S3). Based on kernel density estimation and its 95% confidence interval, the phage resistance threshold was defined as RBG ≥ 0.53 (Fig. S3). Accordingly, 166 isolates were considered resistant to the focal phage and used for subsequent studies, and 14 isolates were discarded due to restored phage sensitivity. We then evaluated cross-resistance patterns using interaction matrices between these isolates and eight phages (Fig. 1B-E). The results showed that resistant strains selected by an individual phage often led to resistance to other phages that target the same receptor (Fig. 1C-E). Resistant strains selected by phages targeting BtuB have little cross-resistance to phages that targeting core and O-antigen (Fig. 1E). However, resistant strains selected by phages targeting the core and O-antigen exhibit cross-resistance with each other, but this cross-resistance is asymmetric. Specifically, 44.7% (34/76) of resistant strains selected by core-targeting phages were also resistant to O-antigen-targeting phages, whereas only 21.6% (8/37) of resistant strains selected by O-antigen-targeting phages were resistant to core-targeting phages (Fig. 1C and D). Overall, the cross-resistance of strains resistant to a specific phage to other phages depends on the type of receptor used by the phage.

### Receptor-associated gene mutations drive cross-resistance through structural disruption

**Figure 2:**
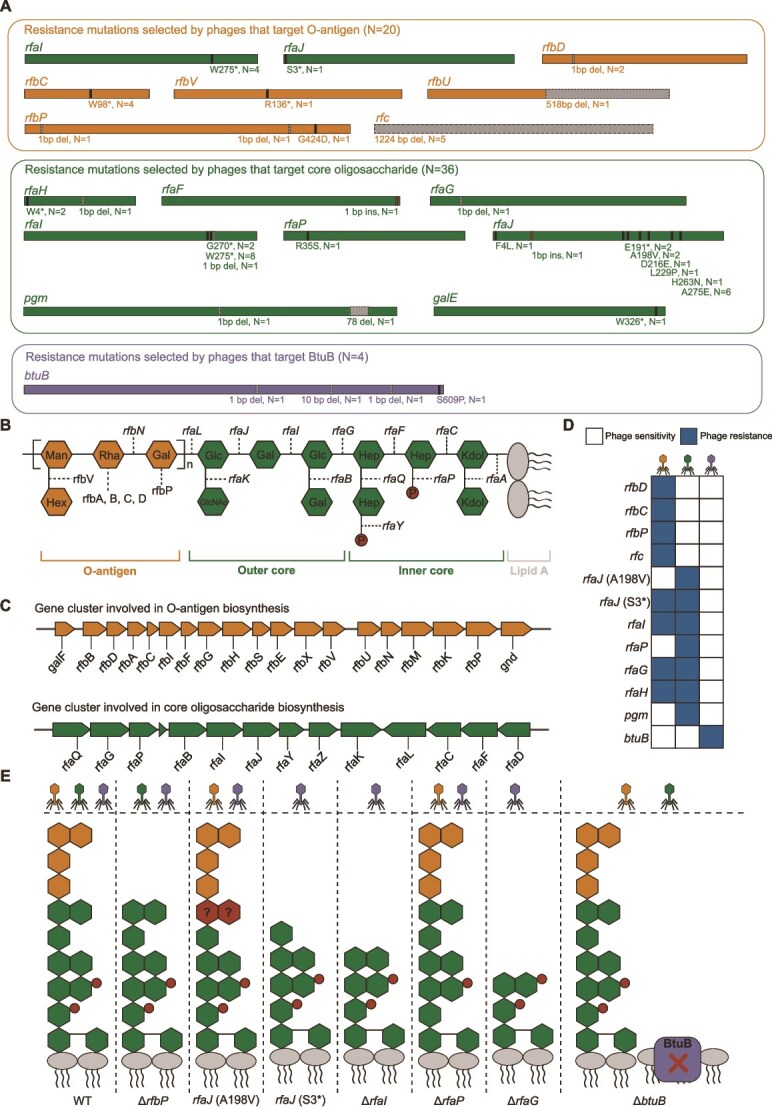
Gene mutations and surface structural changes that mediate phage cross-resistance. (**A**) Mutated genes selected by phages targeting different receptors. Orange represents genes associated with O-antigen truncation, green represents genes associated with core truncation, and purple represents the gene encoding BtuB protein. (**B**) General structure of LPS showing the genes required for its biosynthesis. (**C**) The *rfb* and *rfa* gene clusters involved in the biosynthesis of O-antigen and core. (**D**) The range of phage resistance caused by gene mutations. (**E**) The range of phage resistance caused by bacterial surface structural changes. Phages that can infect specific surface structures are listed at the top. Yellow phage target O-antigen, green phage target the core, and purple phage target BtuB.

To investigate the genetic basis of bacterial cross-resistance against phages, we performed WGS on 59 phage-resistant strains (Table S4). These strains were grouped based on the phage target specificity that selected for resistance: core (N = 35), O-antigen (N = 20), and BtuB (N = 4). Our results indicate that bacteria often develop resistance by acquiring mutations in receptor biosynthesis genes under selective pressure from specific phages. All phage-resistant strains exhibited mutations in receptor-related genes (Table 2, Fig. 2A), with five strains additionally carrying mutations in non-receptor genes (*pabC*, *selB*, *tktA*, and *wcaD*). Regarding receptor-associated mutations, resistance selected by phages targeting BtuB exclusively involved mutations in the *btuB* gene. In contrast, resistance selected by phages targeting LPS involved mutations in 14 distinct genes associated with LPS biosynthesis (Fig. 2A). This difference arises because LPS biosynthesis is a highly complex process, involving numerous enzymes and transporters spanning the cytoplasm, inner membrane, and periplasmic space [53]; mutations in genes governing these processes can confer resistance to LPS-targeting phages by altering LPS structure. Resistance mutations selected by phages targeting either LPS or BtuB specifically disrupted binding only to their respective host receptors, thereby explaining the observed absence of cross-resistance between these two groups (Fig. 1C-E).

**Table 2:** Receptor-associated mutations of phage-resistant clones.

Function	Gene	Resistance mutation
Gene cluster involved in O-antigen biosynthesis	*rfbD*	1 bp del.
	*rfbC*	W98^*^
	*rfbV*	R136^*^
	*rfbU*	824 bp del.
	*rfbP*	1 bp del.
	*rfbP*	G424D
Link O-antigen repeat unit	*rfc*	2383 bp del.
Gene cluster involved in core oligosaccharide biosynthesis	*rfaF*	1 bp ins.
	*rfaJ*	S3^*^
	*rfaJ*	E191^*^
	*rfaJ*	F4L
	*rfaJ*	A198V
	*rfaJ*	L229P
	*rfaJ*	A275E
	*rfaJ*	D216E
	*rfaJ*	H263N
	*rfaJ*	1 bp ins.
	*rfaI*	G270^*^
	*rfaI*	W275^*^
	*rfaI*	1 bp del.
	*rfaP*	R35S
	*rfaG*	1 bp del.
Transcription factors that control lipopolysaccharide biosynthesis	*rfaH*	W4^*^
	*rfaH*	1 bp del.
Phosphoglucomutase	*pgm*	1 bp del.
	*pgm*	78 bp del.
UDP-galactose epimerase	*galE*	W326^*^
Vitamin B_12_ transporter BtuB	*btuB*	1 bp del.
		S609P

^*^indicates a stop codon

The LPS-related mutations included genes in the core biosynthesis gene cluster *rfa*, the O-antigen biosynthesis gene cluster *rfb*, the LPS transcriptional regulator gene *rfaH* [54], and genes involved in synthesizing activated sugar nucleotide precursors required for LPS assembly (*pgm*, *galE*) [55] (Fig. 2A-C). Further analysis of mutated genes selected by phages targeting either the core or O-antigen of LPS revealed distinct mutational preferences. Mutations selected by phages targeting core occurred primarily in the *rfa* gene cluster (29/35), leading to core truncations or modifications that manifested in two resistance patterns. Core truncation (concomitant with O-antigen absence) caused by mutations in genes *rfaI*, *rfaJ* (S3^*^, E191^*^, F4L, H263N), *rfaG*, and *rfaF* conferred resistance to both O-antigen-targeting and core-targeting phages. Loss of phosphate groups due to *rfaP* gene mutation or specific missense mutations in the *rfaJ* gene (A198V, L229P, A275E, D216E) conferred resistance exclusively to core-targeting phages (Fig. 2D-E, Fig. S4A). LPS silver staining indicated that the *rfaJ* gene mutant (A198V) retained partial O-antigen (Fig. S4B), suggesting that only the core structure was altered whereas the O-antigen required for recognition by O-antigen-targeting phages was not disrupted, which explains the different resistance patterns caused by *rfaJ* gene mutations. Mutations in the *pgm* gene and *galE* gene also selected by phages targeting core, conferred resistance specifically to these phages. In contrast, mutations leading to O-antigen truncation or loss were primarily selected by phages targeting the O-antigen (15/20). These included mutations in the *rfb* cluster (10/20) and the *rfc* gene (5/20), which exclusively conferred resistance to O-antigen-targeting phages. Additionally, these phages selected mutations causing core truncation in the *rfa* cluster (5/20), which conferred resistance to both O-antigen-targeting and core-targeting phages (Fig. 2A). Therefore, the mutational patterns selected by phages that target core and O-antigen explain the asymmetric cross-resistance (Fig. 2B-D).

In summary, we elucidated the cross-resistance mechanisms of phage-resistant strains (selected by phages targeting O-antigen, core, or BtuB) against other phages through correlation of mutations conferring specific phage-resistant phenotypes to alterations in bacterial surface structures (Fig. 2D-E).

### Mutations in receptor-related genes dominate phage resistance and their fitness costs

**Figure 3:**
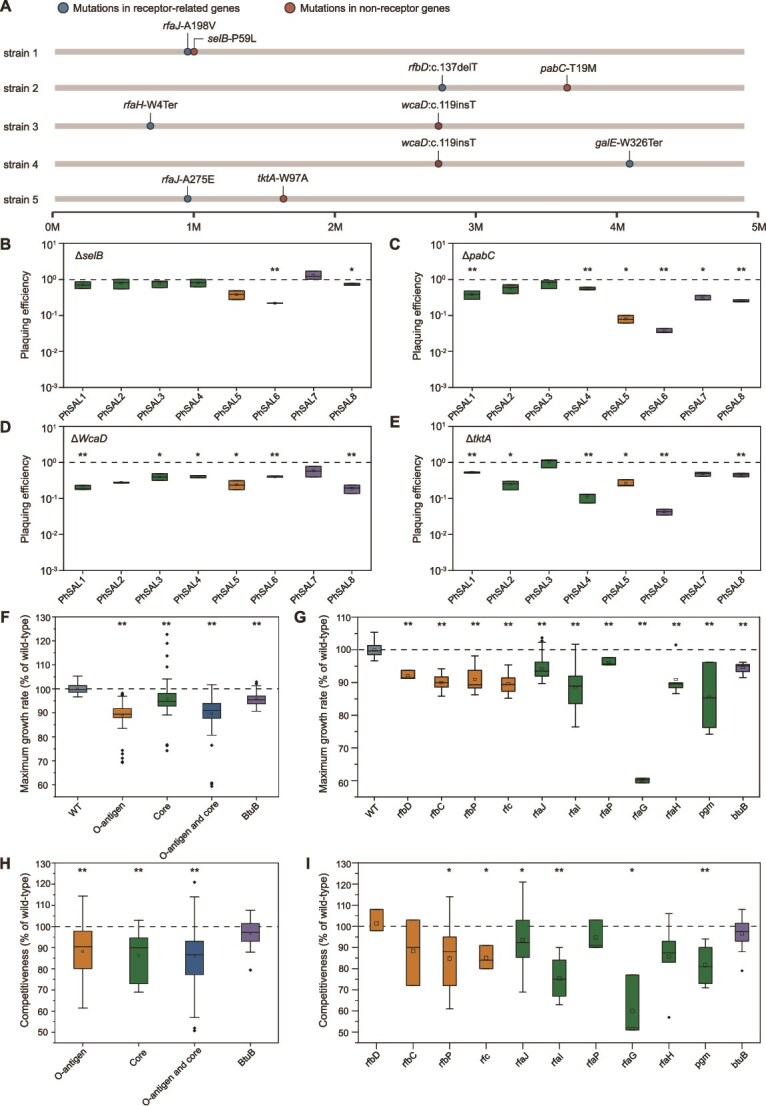
Contribution of non-receptor gene mutations in phage resistance and the fitness costs associated with phage resistance. (**A**) Graphical presentation of five phage-resistant strains carrying mutations in both receptor-related and non-receptor genes. (**B-E**) EOP of eight phages on Δ*selB* (**B**), Δ*pabC* (**C**), Δ*WcaD* (**D**), and Δ*tktA* (**E**) deletion strains relative to WT bacteria. The dotted line represents the WT bacterial control baseline. Statistical significance compared to WT bacteria was assessed using unpaired Student’s t-test (^*^  *P* ≤ 0.05, ^**^  *P* ≤ 0.01). (**F-G**) Maximum growth rates of phage-resistant strains with different resistance ranges (N = 100) (**F**) and single-gene mutations (N = 52) (**G**) relative to wild-type (WT) bacteria. Statistical significance was assessed using Mann–Whitney U tests (^*^  *P* ≤ 0.05, ^**^  *P* ≤ 0.01). (**H-I**) Competitive ability of phage-resistant strains with different resistance ranges (N = 27) (**H**) and single-gene mutations (N = 27) (**I**) relative to WT strain. Statistical significance was assessed using a one-sample t-test (^*^  *P* ≤ 0.05, ^**^  *P* ≤ 0.01). Box plots display the median (center line), mean (squares), interquartile range (boxes), and outliers (dots).

As mentioned earlier, we found five phage-resistant strains carrying mutations in both receptor-related and non-receptor genes (Fig. 3A). To elucidate the contribution of these non-receptor gene mutations to the phage resistance phenotypes, we constructed gene deletion strains (Δ*selB*, Δ*pabC*, Δ*WcaD*, and Δ*tktA*) and quantitatively evaluated their effects on phage infectivity through EOP (Fig. 3B-E). Unlike phage receptor gene deletions which abolished plaque formation (Fig. S2A), these non-receptor gene deletions only slightly reduced the EOP of some phages. Across the four deletion strains, Δ*selB* significantly decreased EOP to 0.22–0.73, Δ*pabC* to 0.04–0.55, Δ*wcaD* to 0.19–0.42, and Δ*tktA* to 0.04–0.52. This indicates that although non-receptor gene mutations contribute to phage resistance, the primary determinants factors remain mutations in receptor-associated genes, consistent with previous studies [56]. The coexistence of these four non-receptor gene mutations with receptor-related mutations suggests that bacteria infected with phages might first acquire non-receptor gene mutations. However, because these mutations failed to confer complete resistance, the bacteria acquired subsequently undergo receptor-related gene mutations.

Because LPS maintains bacterial structural integrity [53] and the OMP BtuB facilitates the uptake of vitamin B_12_ essential for bacterial growth [57], acquiring phage resistance through LPS structural variation or BtuB mutation may impose a fitness cost. The isolated phage-resistant strains can be classified into four categories according to their resistance phenotypes: (i) resistance to O-antigen-targeting phages, (ii) resistance to core-targeting phages, (iii) resistance to both O-antigen and core-targeting phages, and (iv) resistance to BtuB-targeting phages. We selected 100 resistant strains (25 strains of each resistance phenotype) to investigate the correlation between the range of bacterial cross-resistance to phages and fitness trade-offs. The maximum bacterial growth rate served as an indicator to assess fitness cost, with results showing that the fitness of the above four resistance phenotypes relative to the WT strain was 89.1%, 95.8%, 89.7%, and 95.7%, respectively (Fig. 3F). The fitness costs did not increase with the range of phage resistance: resistance to both O-antigen-targeting and core-targeting phages exhibited comparable fitness reduction to resistance to O-antigen-targeting phages alone (Fig. 3F). Given that distinct gene mutations could confer the same phage resistance phenotypes (Fig. 2D), we further quantified fitness costs in phage-resistant strains with single-gene mutations (N = 52). The results revealed significant fitness cost differences among single-gene mutations (*rfaI*, *rfaJ*, *rfaG*, and *rfaH*) conferring resistance to both O-antigen-targeting and core-targeting phages. Relative fitness ranged from 60.3% to 94.3% compared to the WT strain, with the *rfaJ* mutation exhibiting the highest fitness and the *rfaG* mutation the lowest. In contrast, single-gene mutations conferring resistance only to either O-antigen-targeting phages (*rfbD*, *rfbC*, *rfbP*, *rfc*) or core-targeting phages (*rfaP*, *pgm*) showed relatively narrow ranges of fitness cost, from 89.6% to 92.1% and 85.6% to 91.0%, respectively (Fig. 3G). Resistance to BtuB-targeting phages was conferred solely by mutations in the *btuB* gene, with the fitness cost determined by this single gene (Fig. 2D, Fig. 3F-G).

The competitive ability of phage-resistant strains relative to WT strain also serves as an important indicator of the fitness cost. We further assessed the relative competitive ability of phage-resistant strains (N = 27). Unlike the growth rate data, competitive fitness based on resistance range was 88.1% for strains resistant to O-antigen-targeting phages, 86.2% for core-targeting phages, and 86.0% for both types (Fig. 3H). Despite this discrepancy at the resistance range level, single-gene mutation analysis revealed a pattern consistent with the growth rate data: distinct gene mutations conferring the same resistance range led to varying degrees of fitness reduction. Among mutations conferring resistance to both O-antigen-targeting and core-targeting phages (*rfaJ*, *rfaI*, and *rfaG*), the *rfaJ* mutant exhibited the highest competitive fitness (93%), as also observed for growth rate (Fig. 3I). Therefore, the fitness cost of phage resistance via receptor modification depends on the specific gene mutation selected by the phage.

### Fitness costs of mutations conferring phage resistance drive differences in phage antibacterial efficacy

**Figure 4:**
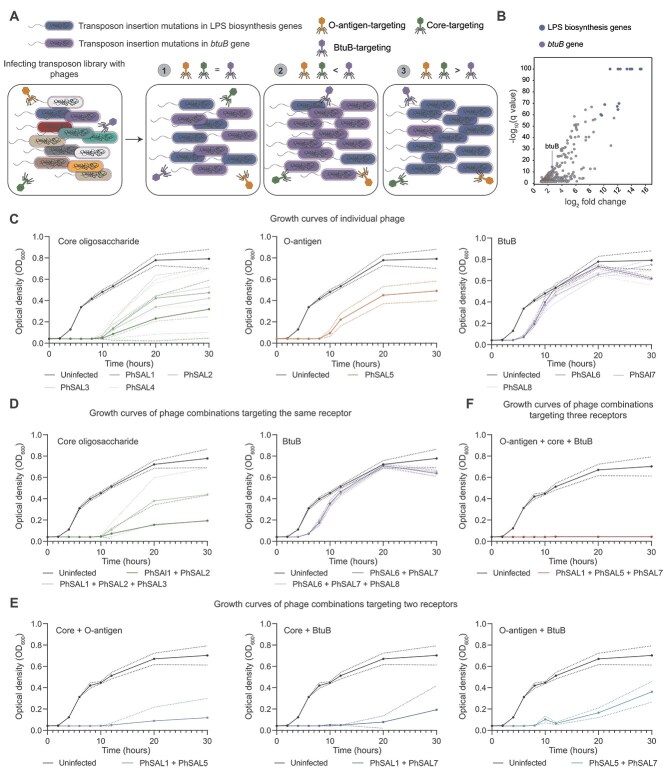
Comparison of antibacterial differences between LPS-targeting and BtuB-targeting phages and evaluation of combination therapy efficacy. (**A**) Legend of three potential outcomes from co-infection of a *Salmonella* transposon mutant library with LPS-targeting phages and a BtuB-targeting phage: i) Comparable abundance of LPS biosynthesis and *btuB* mutants indicates comparable antibacterial efficacy between phage types; ii) Increased *btuB* mutant abundance reflects stronger antibacterial effect of BtuB-targeting phages; iii) Increased LPS biosynthesis mutant abundance reflects stronger antibacterial effect of LPS-targeting phages. (**B**) Scatter plot showing significantly enriched mutants following phage infection, with blue representing LPS biosynthesis gene mutants and purple indicating btuB mutants. (**C-F**) Growth curves for *Salmonella enterica* ATCC 13076 treated with an individual phage (**C**), a combination of phages targeting the same receptor (**D**), a combination of phages targeting two different receptors (**E**), and a combination of phages targeting three different receptors (**F**). Statistical significance was assessed using ANOVA based on AUC followed by Dunnett's T3 test (Table S5).

The fitness cost of the mutant genes selected by phages targeting O-antigen (*rfbC*, *rfbD*, *rfbP*, *rfc*) or core (*rfaI*, *rfaG*, *rfaH*) was generally higher than that of phages targeting BtuB (Fig. 3G). Based on this observed difference in fitness costs, we hypothesized that phages targeting the O-antigen and core region exhibit superior antibacterial effects relative to phages targeting BtuB. To test this hypothesis, we employed transposon insertion sequencing (Tn-seq), in which phage infection of a transposon mutant library led to significant enrichment of receptor gene mutants due to their acquired resistance [58]. We co-infected a *Salmonella* transposon mutant library with phages targeting LPS (PhSAL1 and PhSAL5) and BtuB (PhSAL7). Theoretically, surviving populations would consist mainly of two resistant mutant types: those escaping LPS-targeting phages (LPS biosynthesis gene mutants) and those escaping BtuB-targeting phage (*btuB* mutants). Therefore, changes in the relative abundance of the two resistant mutants in the infected library directly indicate the relative selective pressure imposed by phages during co-infection. If both phages had similar antibacterial effects, the two mutant types would show comparable abundance. If LPS-targeting phages exerted stronger selection, LPS biosynthesis mutants would be more enriched; conversely, greater enrichment of *btuB* mutants would indicate stronger selection by BtuB-targeting phages (Fig. 4A). Tn-seq results showed that mutants with disruptions in LPS biosynthesis genes were the most strongly selected during infection, particularly those in the *rfa* gene cluster (*rfaG*, *rfaQ*, *rfaF*, *rfaJ*, *rfaI*, *rfaK*, and *rfaH*), which exhibited Log_2_FC values ranging from 10.13–15.37 (*q* < 0.001), whereas the enrichment of the *btuB* mutant was negligible (Log_2_FC = 2.11, *q* < 0.001) (Fig. 4B, Table S5). This indicates that in mixed infections, phages targeting LPS exerted absolute dominant selection pressure, exhibiting superior antibacterial effects compared to phages targeting BtuB. To independently verify the differences in phage killing ability, we measured bacterial growth curves under individual phage treatments (Fig. 4C, Table S5). Consistent with the above results, the antibacterial effect of BtuB-targeting phages was significantly lower than that of core-targeting and O-antigen-targeting phages (AUC mean difference = 4.38 and 3.45, respectively, both *P* < 0.001). These quantitative kinetic data corroborate the Tn-seq genetic screening results, together confirming that LPS-targeting phages possess stronger antibacterial activity.

Given that resistant strains selected by phage targeting different receptors exhibited lower cross-resistance, we hypothesized that such combinations might exhibit enhanced antibacterial effects compared to combinations targeting the same receptor. To investigate this, we measured bacterial growth curves for phage combinations targeting the same receptor and different receptors (Fig. 4D-F, Table S5). The results showed that combinations of two or three phages targeting the same receptor exhibited antibacterial effects comparable to those of an individual phage (AUC mean difference range: 0.06–1.39, all *P* > 0.05). In contrast, combinations targeting two different receptors (core and O-antigen, core and BtuB, O-antigen and BtuB) with low or no cross-resistance showed enhanced antibacterial effects compared to individual phages (AUC mean difference range: 1.80–6.19, all *P* < 0.01). However, significant bacterial growth was still observed in these two-receptor combinations (mean AUC range: 1.14–1.89). The combination of targeting three different receptors completely inhibited bacterial growth within 30 h (AUC mean difference = 7.75 compared to control, *P* < 0.001), possibly because the bacteria had either not yet evolved resistance to all three phages simultaneously, or the fitness cost associated with developing resistance to all three phages was prohibitively high.

### Sequential acquisition of resistance to phages targeting different receptors in bacteria

**Figure 5:**
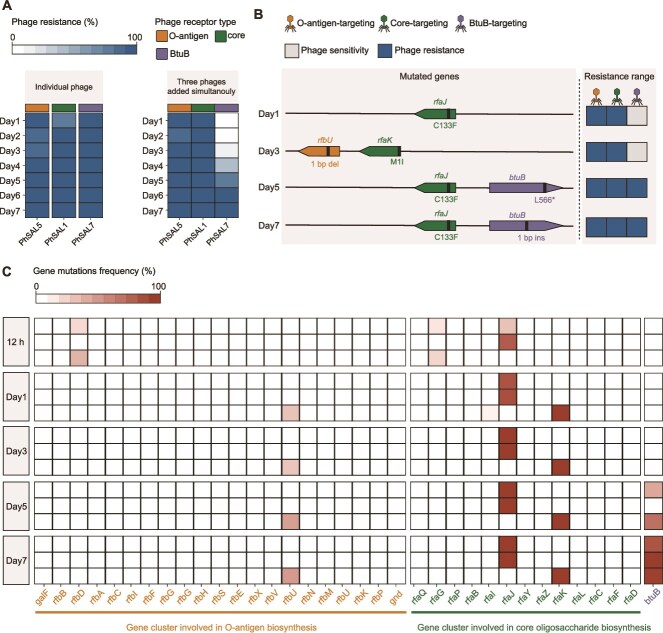
Phage resistance phenotypes and genetic mutations during co-culture of bacteria and phages. (**A**) Phage resistance development rate in bacteria co-cultured with individual phage or a three-phage combination. (**B**) Graphical presentation of genetic mutations and the resistance range in phage-resistant strains at different time points during co-culture with the three-phage combination. (**C**) Heatmap showing temporal changes in population mutation frequencies within LPS biosynthesis genes (*rfa* and *rfb* gene clusters) and the *btuB* gene during co-culture with the three-phage combination.

The genetic basis of bacterial resistance to three different receptor-targeting phages has been elucidated individually, and we now further explore how bacteria simultaneously respond to mixed infection by all three receptor-targeting phages. We selected one effective phage representative targeting each receptor type: PhSAL1 (core-targeting), PhSAL5 (O-antigen-targeting), and PhSAL7 (BtuB-targeting). Serial passaging culture of host bacteria was then performed for 7 days in the presence of each individual phage and a combination of all three phages. From each treatment group, 10 surviving bacterial colonies were isolated daily and tested for sensitivity to the three receptor-targeting phages to quantify the rate of phage resistance evolution. Additionally, whole-population genome sequencing of the three-phage combination treatment group was performed at 12 h and on Days 1, 3, 5, and 7 of co-culture to investigate the temporal evolution of mutations associated with phage resistance. The results of phage resistance phenotype showed that bacteria co-cultured with an individual phage rapidly developed phage resistance within the first day (90%–100%). In contrast, the three-phage treatment delayed the emergence of resistance to the BtuB-targeting phage, with only 10% resistance rate observed on Day 3 (Fig. 5A, Table S5). Within the three-phage treatment group, an arms race dynamic was observed, with the bacterial population broadening its resistance range over time under selective pressure. Resistance to the core-targeting and O-antigen-targeting phages emerged first, reaching 80% for PhSAL1 and 100% for PhSAL5 by Day 1, and both remained at 100% from Day 2 onward. This was followed by resistance to the BtuB-targeting phage (PhSAL7). Complete resistance to all three phages developed by Day 6 (Fig. 5A, Table S5). Across the time course, resistance to LPS-targeting phages consistently arose prior to resistance to the BtuB-targeting phage, possibly due to their greater antibacterial activity.

Whole-population genome sequencing showed that bacteria in the three-phage treatment group primarily acquired resistance mutations in receptor-related genes throughout the co-culture process, along with low-frequency mutations in other genes (Fig. 5C, Fig. S5). At 12 h, mutations in multiple LPS biosynthesis-related genes were detected across all three replicates, including *rfaG*, *rfaJ*, *rfbD*, *pgm*, and *hldE*, with frequencies ranging from 0.095 to 0.822. By Day 1, the mutation patterns stabilized, with *rfaJ* dominating in two replicates (87.2% and 89.6%), conferring resistance to both O-antigen-targeting and core-targeting phages, whereas the third replicate was dominated by *rfbU* (28.9%) and *rfaK* (100%), conferring resistance to O-antigen-targeting and core-targeting phages, respectively. From Day 3 to Day 7, *rfaJ* continued to dominate in the same two replicates, consistently at 100% frequency (Fig. 5C). Throughout the co-culture period, mutations in non-receptor-related genes consistently remained below 20% frequency (Fig. S5). The dominance of receptor-related mutations can be attributed to their decisive role in conferring phage resistance (Fig. 3A-E). Therefore, unlike the diverse mutant genes selected on solid plates, those selected in liquid culture were limited. Among the mutant genes (*rfaJ*, *rfaI*, *rfaG*, and *rfaH*) that confer resistance to both O-antigen-targeting and core-targeting phages, the *rfaJ* gene mutation exhibits the lowest fitness cost (Fig. 3G). This suggests that mutations with low fitness costs are favored during the evolution of phage resistance.

Mutations in the *btuB* gene that confer resistance to BtuB-targeting phage accumulated by Day 5, reaching an average mutation rate of 95.3% by Day 7 (Fig. 5C). Across the three replicates, mutations in LPS biosynthesis genes became dominant first, followed by the accumulation of *btuB* mutations, reflecting a process in which resistance to the different phages was acquired through sequential selection, as supported by the isolation of resistant strains at different time points (Fig. 5B). To further validate this sequential acquisition pattern, we tested another three-phage combination (PhSAL2, PhSAL5, and PhSAL8) targeting different receptors in serial passaging culture with host bacteria. Similarly, bacteria first developed resistance to O-antigen-targeting and core-targeting phages, and ultimately to BtuB-targeting phage, although resistance developed at different rates (Fig. S6A). WGS of resistant isolates revealed that sequential mutations in *rfaJ* and *btuB* conferred resistance to all three phages (Fig. S6B). Therefore, based on phage resistance phenotypes, gene mutations in individual strains, and population-level mutation frequencies, we elucidated the process of evolved phage resistance in bacteria. Under phage selection, mutations conferring lower fitness costs in receptor-associated genes are enriched. The sequential enrichment of mutations in different receptor-encoding genes ultimately enables the population to resist all phages targeting these receptors.

## Discussion

**Figure 6:**
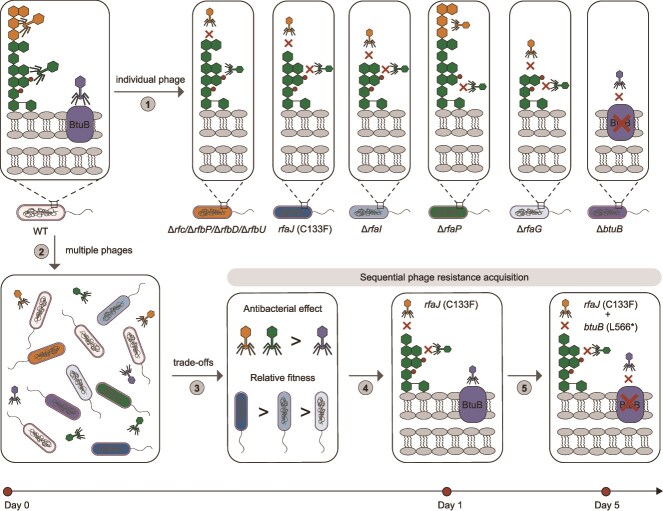
Schematic diagram of phage resistance patterns and evolutionary trade-offs. Under individual phage pressure, *Salmonella* acquires resistance to LPS-targeting or BtuB-targeting phages through LPS truncation or BtuB mutations, respectively. Under simultaneous phage pressure, driven by differential fitness costs of mutations and phage inhibitory strengths, *Salmonella* evolves resistance preferentially against the strongly inhibitory LPS-targeting phage. This occurs through low-fitness-cost *rfaJ* gene mutations, followed by *btuB* gene mutations conferring resistance to BtuB-targeting phages.

The highly specific interaction between phage and host bacteria relies on the phage binding to receptors on the bacterial surface. Here, we used *S. enterica* ATCC 13076 and eight newly isolated phages targeting three different receptors (O-antigen, core oligosaccharide, BtuB) to investigate phage cross-resistance patterns and evolutionary trade-offs under pressure from individual phages or phage combinations. The results showed that cross-resistance of resistant strains selected by an individual phage to other phages is receptor-dependent. *Salmonella* developed resistance to O-antigen-targeting or core-targeting phages through diverse LPS truncations, and the fitness cost associated with phage resistance mutations is gene-specific. Under pressure from a combination of three phages targeting different receptors, resistance to LPS-targeting phages is first selected. Among mutations conferring phage resistance, those in the *rfaJ* gene with the lowest fitness cost dominate. Subsequently, resistance to BtuB-targeting phages emerges through accumulation of *btuB* gene mutations (Fig. 6). Therefore, under selective pressure from diverse phages, low-fitness-cost mutations and sequential resistance acquisition are selected, enabling bacteria to resist simultaneous infection. Our findings elucidate the pivotal role of phage receptor type in phage cross-resistance and resistance-associated fitness trade-offs, and reveal how bacterial populations evolve to escape diverse phage predation.

LPS is a common receptor for phage adsorption. Although previous studies have demonstrated that phage resistance evolved by bacteria under pressure from LPS-targeting phages can confer varying levels of cross-resistance to other LPS-targeting phages [41], the underlying factors responsible for this differential cross-resistance remain unexplained. We further subdivided LPS-targeting phages into those targeting the O-antigen or core regions. Analysis of cross-resistance networks combined with WGS revealed that these two phage types selected distinct resistance mutation patterns in bacteria, resulting in asymmetric cross-resistance (Fig. 1C-D, Fig 2A-D). The LPS structure is critical for bacterial colonization and immune evasion within hosts [59, 60]. Consequently, phage-driven structural variations in LPS may impact the survival fitness of phage-resistant strains within the host. Furthermore, LPS truncation increases antibiotic susceptibility, and combining LPS-targeting phages with antibiotics can delay the emergence of phage resistance [61]. Our results elucidate the resistance pathways selected by O-antigen-targeting and core-targeting phages, enhancing understanding of the trade-off between phage resistance and bacterial immune evasion, and providing insights for the therapeutic design of phage-antibiotic combinations. Screening of host factors required for phage infection on a genome-wide scale and genetic analysis of spontaneous phage-resistant strains indicate that host bacteria may confer phage resistance through non-receptor-related gene mutations [62, 63]. This study quantified the level of phage resistance conferred by non-receptor-related single-gene deletion strains (Δ*selB*, Δ*pabC*, Δ*WcaD*, and Δ*tktA*) (Fig. 3B-E), and tracked the frequency of gene mutations during the evolution of phage resistance through whole-population genome sequencing (Fig. 5B), indicating that host bacteria mainly achieve defense against phages through receptor modification during co-evolution.

The fitness costs associated with phage resistance underscore the pivotal role of receptor specificity and mutant genes. Serving as the major outer membrane component in most Gram-negative bacteria, LPS plays a crucial role in protecting bacteria against environmental stressors, including phages, antibiotics, and host immune responses [64, 65]. BtuB is an OMP in bacteria that mediates TonB-dependent uptake of vitamin B_12_ (cobalamin) [57]. To assess the fitness cost associated with acquiring resistance to phages targeting LPS (either O-antigen or core or both) and to phages targeting BtuB from the perspective of bacterial population evolution, we selected maximum growth rate (reflecting basal metabolic efficiency) and competitive ability (revealing population dynamics under resource limitation) [66] as metrics, which are widely employed to quantify fitness costs [10, 42, 67]. We found fitness costs of mutations conferring same resistance phenotypes vary significantly by gene (Fig. 3F-I). This finding may extend to fitness costs associated with resistance against other phages that utilize macromolecules or proteins whose expression is regulated by multiple genes as receptors (such as capsules and flagella) [68, 69]. The heterogeneity in gene-specific fitness costs can be leveraged to predict bacterial population dynamics during phage therapy, including evolutionary trajectories and dominant resistant subpopulations. For studies investigating either bacterial virulence or phage-antibiotic combination therapy, it is imperative to assess whether resistance to phages targeting different receptors results in attenuated virulence and increased antibiotic susceptibility, and to quantify the magnitude of these phenotypic changes.

Previous studies on the antibacterial efficacy of phages have overlooked the influence of receptor targeting [70, 71]. Here, we examined the relationship between the fitness costs associated with phage resistance selected by phages targeting different receptors and antibacterial efficacy. Our results showed that receptor-associated mutations selected by LPS-targeting phages impose higher fitness costs and exhibited superior antibacterial efficacy compared to those selected by BtuB-targeting phages (Fig. 4A-C). The antibacterial efficacy of phage combinations targeting the same receptor showed no enhancement over that of individual phages (Fig. 4D). In contrast, combinations targeting two different receptors indicated that although O-antigen-targeting and core-targeting phages exhibited cross-resistance, their combined antibacterial activity remained superior to their respective combinations with BtuB-targeting phages (Fig. 4E). This suggests that when designing phage cocktails, both the antibacterial efficacy of phages targeting different receptors and the extent of cross-resistance mediated by receptor specificity should be considered. Furthermore, the combination simultaneously targeting all three receptors achieved complete and sustained suppression (Fig. 4F), demonstrating that phage cocktails targeting different receptors on the same host represent a robust design strategy.

By investigating the evolutionary dynamics of phage resistance under the selective pressure of a three-phage combination targeting different receptors, we have elucidated phage resistance evolution strategies driven by phage receptor specificity. All three replicates of the three-phage treatment group first developed resistance to the LPS-targeting phages through mutations in LPS biosynthesis genes (Fig. 5C), because these phages exerted stronger selection pressure (Fig. 4A-C). Resistance to the BtuB-targeting phage was subsequently achieved through the accumulation of mutations in the *btuB* gene (Fig. 5C). The sequential enrichment of mutations in these receptor-related genes reflects sequential selection for resistance to phages targeting different receptors, providing insight into the evolutionary dynamics governing bacterial evasion of phage predation in microbial communities. Furthermore, previous studies have shown that although the resistance mechanisms (receptor modification, CRISPR immunity) selected by bacteria during co-culture with phages depend on specific environmental conditions (nutrient availability and community composition), they all involve mutations with low fitness cost [14, 72, 73]. In this study, after 12 h of co-culture with a three-phage combination containing LPS-targeting phages, *Salmonella* developed diverse resistance-conferring mutations in genes such as *rfaG*, *rfaJ*, and *rfbD*. However, the *rfaJ* gene mutation with the lowest fitness cost became the dominant one in the later stage of co-culture (Fig. 5C). This outcome indicates a competition for survival among resistant mutants, resulting in the prevalence of those with low-fitness-cost resistance mutations. Therefore, during the sequential acquisition of phage resistance, bacterial populations predominantly accumulate genetic mutations that incur low fitness costs.

In conclusion, our study characterizes a receptor-dependent phage cross-resistance pattern and reveals how low-cost, sequential resistance acquisition is selected under pressure from diverse phages targeting different receptors. These findings provide insights into the impact of phages on bacterial evolution within microbial communities and contribute to the rational design of combinatorial phage therapies.

## Supplementary Material

Supplementary-Material_wrag077

## Data Availability

The sequencing data of *Salmonella* phages were submitted to GenBank under BioProject number PRJNA1299103 (reviewer link: https://dataview.ncbi.nlm.nih.gov/object/PRJNA1299103?reviewer=2r8tmvel57nqescpv0cfm30q2p). The assembled complete *Salmonella* phages genomes were deposited to GenBank under accession numbers PV988430-PV98437 (released after publication). The sequencing data of phage-resistant strains and *Salmonella* populations were submitted to GenBank under BioProject number PRJNA1299054 (reviewer link: https://dataview.ncbi.nlm.nih.gov/object/PRJNA1299054?reviewer=usngr6foed10itcdpe5e9sjeiu). The Tn-seq sequencing data were submitted to GenBank under BioProject number PRJNA1400527 (reviewer link: https://dataview.ncbi.nlm.nih.gov/object/PRJNA1400527?reviewer=egceojm50m362e57kh1f4vuedg).

## Code availability

The code supporting the findings of this paper is available in the online repository: https://github.com/Yingting-Wu/Phage-resistance
