# Supplementary material for: Phage receptor specificity drives cross-resistance patterns and governs fitness trade-offs during sequential resistance acquisition in Salmonella
Source: ISME J. 2026 Apr 11;20(1):wrag077. doi: 10.1093/ismejo/wrag077 (PMC13196588; doi:10.1093/ismejo/wrag077)
Supplement: Supplementary-Material_wrag077 [file supplementary-material_wrag077.zip › Fig_S4_wrag077.pdf]

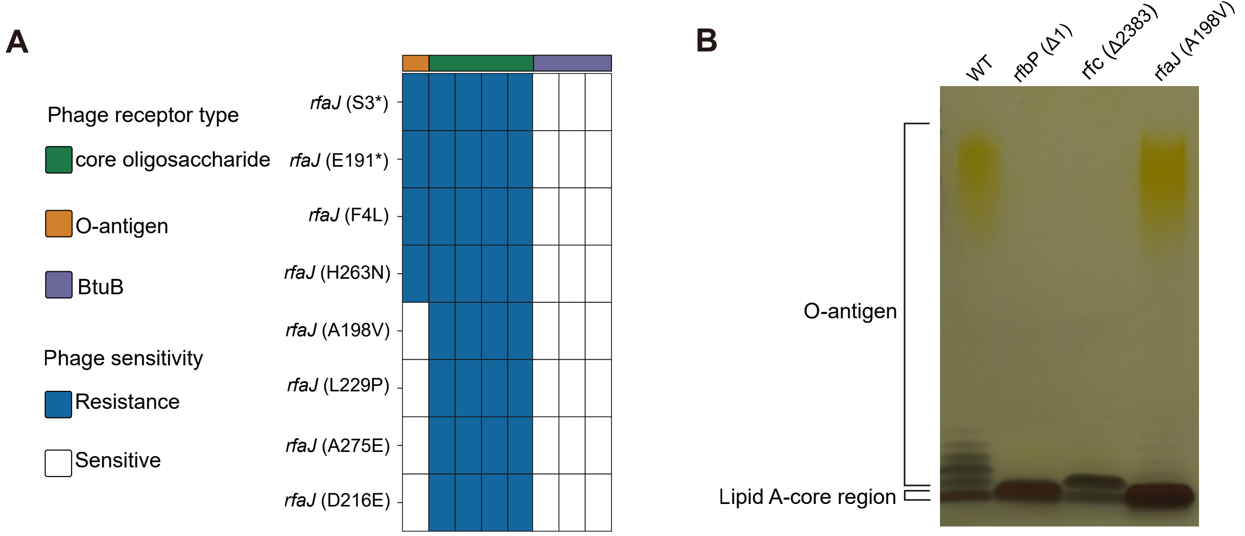
A
Phage receptor type
core oligosaccharide
O-antigen
BtuB
Phage sensitivity
Resistance
Sensitive
rfaJ (S3*)
rfaJ (E191*)
rfaJ (F4L)
rfaJ (H263N)
rfaJ (A198V)
rfaJ (L229P)
rfaJ (A275E)
rfaJ (D216E)
B
WT
rfbP (Δ1)
rfc (Δ2383)
rfaJ (A198V)
O-antigen
Lipid A-core region
