# Supplementary material for: Phage receptor specificity drives cross-resistance patterns and governs fitness trade-offs during sequential resistance acquisition in Salmonella
Source: ISME J. 2026 Apr 11;20(1):wrag077. doi: 10.1093/ismejo/wrag077 (PMC13196588; doi:10.1093/ismejo/wrag077)
Supplement: Supplementary-Material_wrag077 [file supplementary-material_wrag077.zip › Fig_S5_wrag077.pdf]

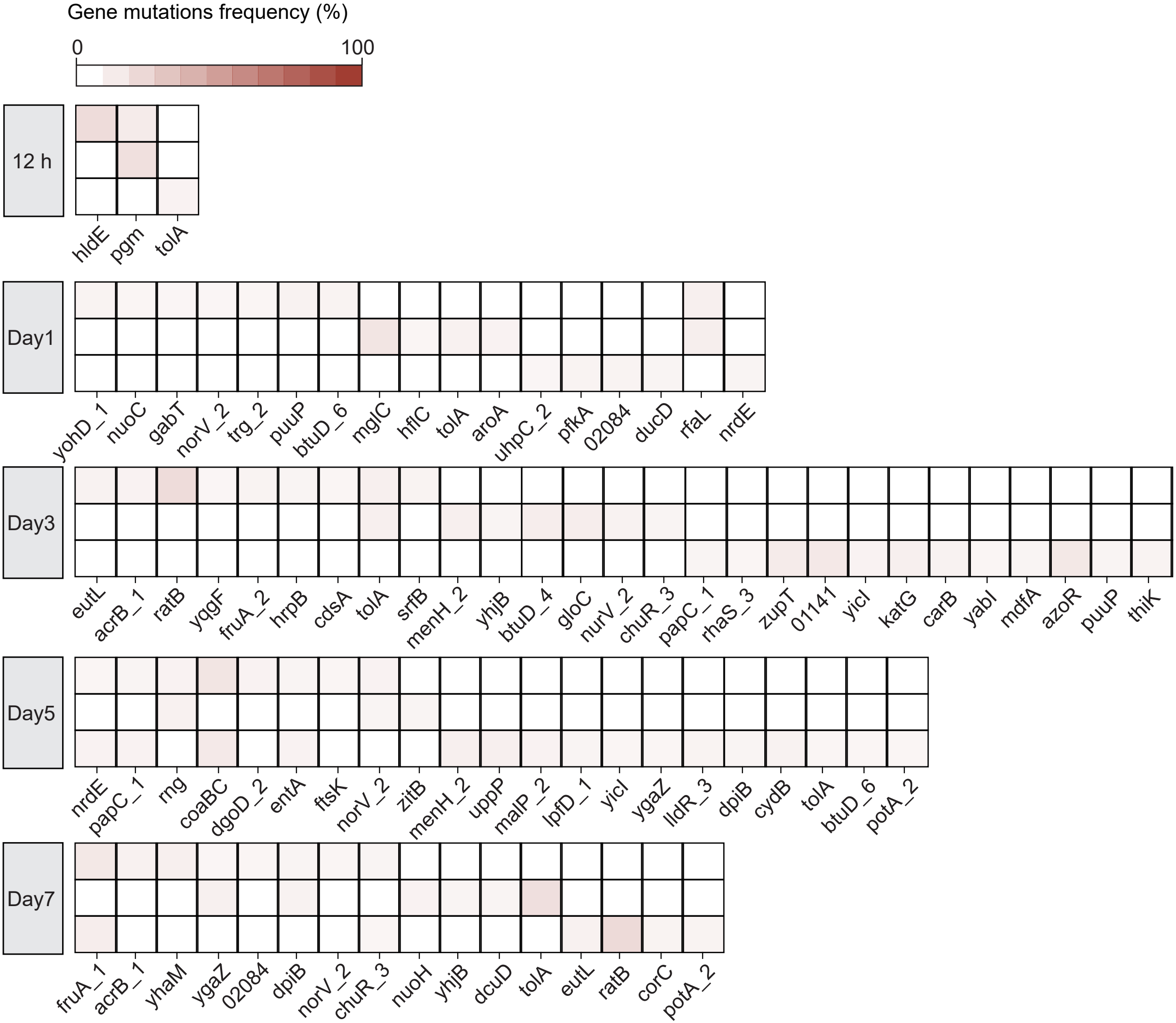
Gene mutations frequency (%)
0
100
12 h
hldE
pgm
tolA
Day1
yohD_1
nuoC
gabT
norV_2
trg_2
puuP
btuD_6
mglC
hflC
tolA
aroA
uhpC_2
pfkA
02084
ducD
rfaL
nrdE
Day3
eutL
acrB_1
ratB
yqgF
fruA_2
hrpB
cdsA
tolA
srfB
menH_2
yhjB
btuD_4
gloC
nurV_2
chuR_3
papC_1
rhaS_3
zupT
01141
yicI
katG
carB
yabI
mdfA
azoR
puuP
thiK
Day5
nrdE
papC_1
rng
coaBC
dgoD_2
entA
ftsK
norV_2
zitB
menH_2
uppP
malP_2
lpfD_1
yicI
ygaZ
lldR_3
dpiB
cydB
tolA
btuD_6
potA_2
Day7
fruA_1
acrB_1
yhaM
ygaZ
02084
dpiB
norV_2
chuR_3
nuoH
yhjB
dcuD
tolA
eutL
ratB
corC
potA_2
