# Supplementary material for: Phage receptor specificity drives cross-resistance patterns and governs fitness trade-offs during sequential resistance acquisition in Salmonella
Source: ISME J. 2026 Apr 11;20(1):wrag077. doi: 10.1093/ismejo/wrag077 (PMC13196588; doi:10.1093/ismejo/wrag077)
Supplement: Supplementary-Material_wrag077 [file supplementary-material_wrag077.zip › Fig_S6_wrag077.pdf]

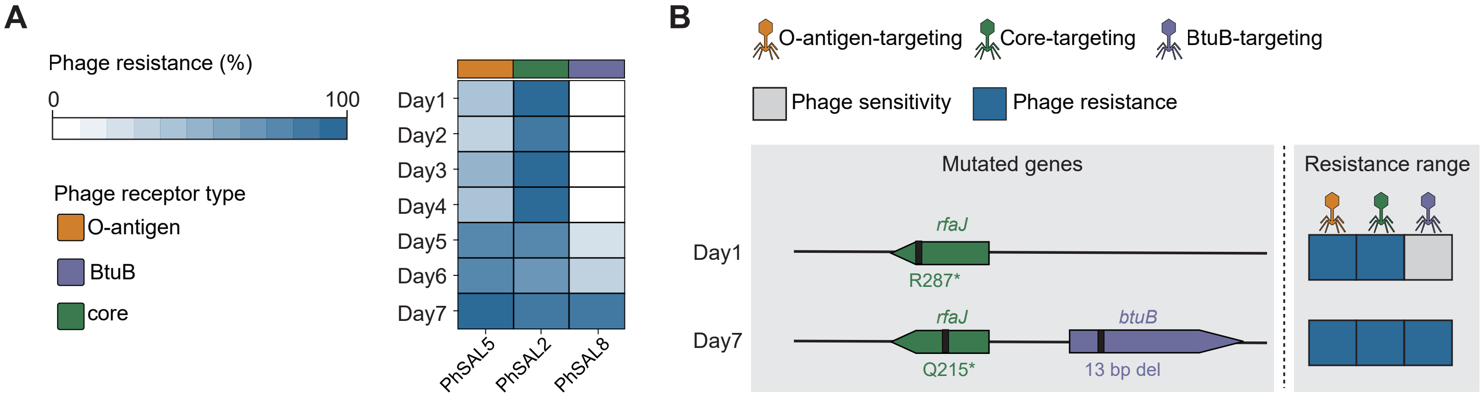
A
Phage resistance (%)
0
100
Phage receptor type
O-antigen
BtuB
core
Day1
Day2
Day3
Day4
Day5
Day6
Day7
PhSAL5
PhSAL2
PhSAL8
B
O-antigen-targeting
Core-targeting
BtuB-targeting
Phage sensitivity
Phage resistance
Mutated genes
Resistance range
Day1
rfaJ
R287*
Day7
rfaJ
Q215*
btuB
13 bp del
