# Supplementary material for: Phage receptor specificity drives cross-resistance patterns and governs fitness trade-offs during sequential resistance acquisition in Salmonella
Source: ISME J. 2026 Apr 11;20(1):wrag077. doi: 10.1093/ismejo/wrag077 (PMC13196588; doi:10.1093/ismejo/wrag077)
Supplement: Supplementary-Material_wrag077 [file supplementary-material_wrag077.zip › Supplementary_legends_wrag077.docx]

**Supplementary figure legends**

**Figure. S1 Genome analysis and receptor identification of *Salmonella* phages. A** Phylogenetic tree constructed based on the major capsid proteins of eight isolated *Salmonella* phages and homologous proteins. Red represents phages isolated in this study. **B** Genomic alignment of *Salmonella* phages. **C** Transmission electron micrograph of Isolated *Salmonella* phages, scale bar = 50 nm.

**Figure. S2 Receptor identification of *Salmonella* phages. A** EOP of eight phages on 14 deletion strains (Δ*rfaP*, Δ*rfaC*, Δ*rfaY*, Δ*rfaF*, Δ*rfaQ*, Δ*rfaG*, Δ*rfaB*, Δ*rfaI*, Δ*rfaJ*, Δ*rfaK*, Δ*rfbP*, Δ*OmpC*, Δ*btuB*, Δ*fliC*) relative to WT strain. The dotted line represents the WT bacterial control baseline, and “bd” on the dashed line indicates below the limit of detection. **B** The adsorption rates of phages on deletion strains relative to the WT strain. Phages identified as LPS-targeting in EOP assays were selected for adsorption rate measurements using Δ*rfaC* and Δ*rfbP* deletion strains, whereas phages targeting BtuB were assessed using the Δ*btuB* deletion strain. Statistical significance was assessed using unpaired Student’s t-test (* *P* ≤ 0.05, ** *P* ≤ 0.01).

**Figure. S3 RBG Kernel Density Curve with Threshold and 95% Confidence Interval.** The red dashed line indicates the initial threshold of 0.511, determined through kernel density estimation and the peak-valley method, with the semi-transparent red rectangle representing its 95% confidence interval [0.49, 0.53].

**Figure. S4 Effects of *rfaJ* gene mutations on the range of phage resistance and LPS structure. A** The range of phage resistance conferred by different mutations in the *rfaJ* gene. **B** Comparative LPS profiles of WT bacteria and phage-resistant mutant strains. WT bacteria exhibited complete LPS structures, *rfbP* and *rfc* gene mutants lacked O-antigen, and *rfaJ* (A198V) gene mutant retained part of the O-antigen.

**Figure. S5 Heatmap showing low-frequency mutations in the bacterial population during co-culture with the three-phage combination.**

**Figure. S6** **Phage resistance phenotypes and genetic mutations during co-culture of bacteria and another three-phage combination (PhSAL2, PhSAL5, PhSAL8). A** Phage resistance development rate in bacteria co-cultured with the three-phage combination. **B** Graphical presentation of genetic mutations and the resistance range in phage-resistant strains on day 1 and 7 of co-culture.

**Supplementary table legends**

Table S1: Primer sequences used to construct gene deletion strains

Table S2: Intergenomic similarity and ANI among *Salmonella* phages

Table S3: RBG values for all isolates

Table S4: Genetic mutations in phage-resistant strains and *Salmonella* populations

Table S5: Raw data and test statistic in statistical analysis
