# Supplementary figures and images for: Phage receptor specificity drives cross-resistance patterns and governs fitness trade-offs during sequential resistance acquisition in Salmonella
Source: ISME J. 2026 Apr 11;20(1):wrag077. doi: 10.1093/ismejo/wrag077 (PMC13196588; doi:10.1093/ismejo/wrag077)

**A**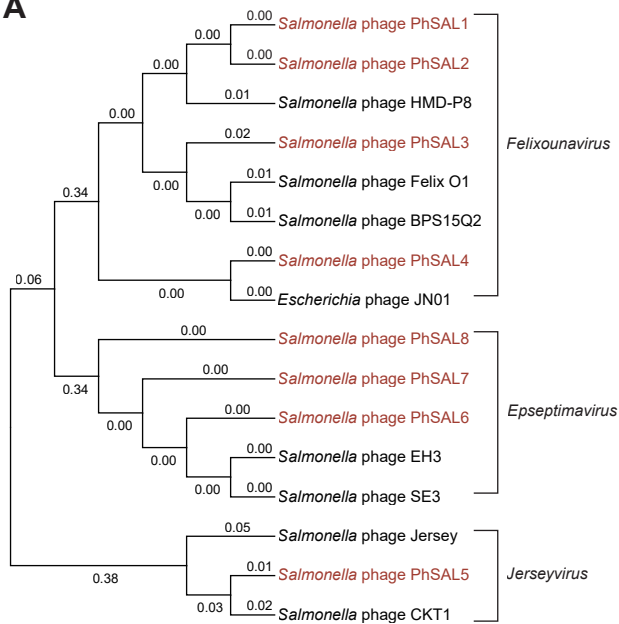**B**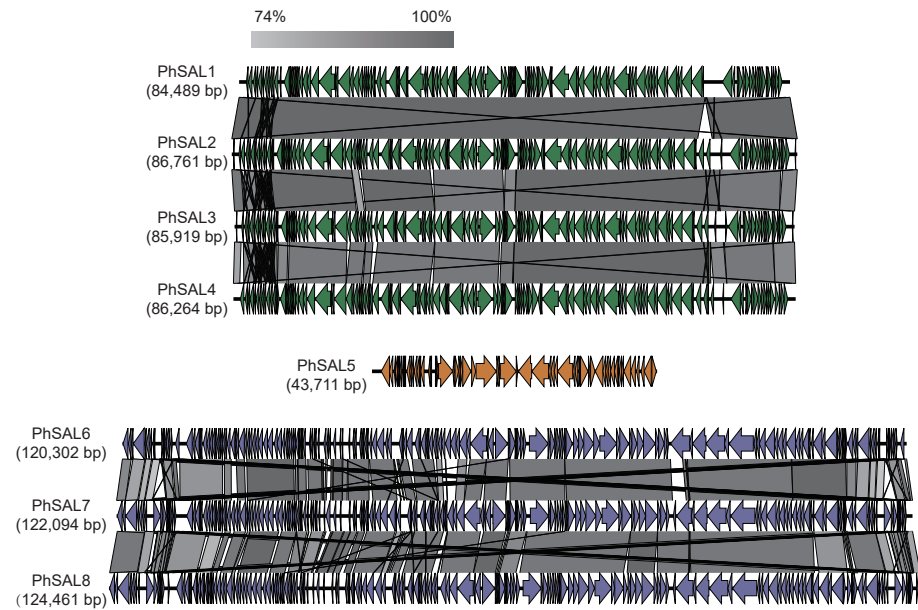**C**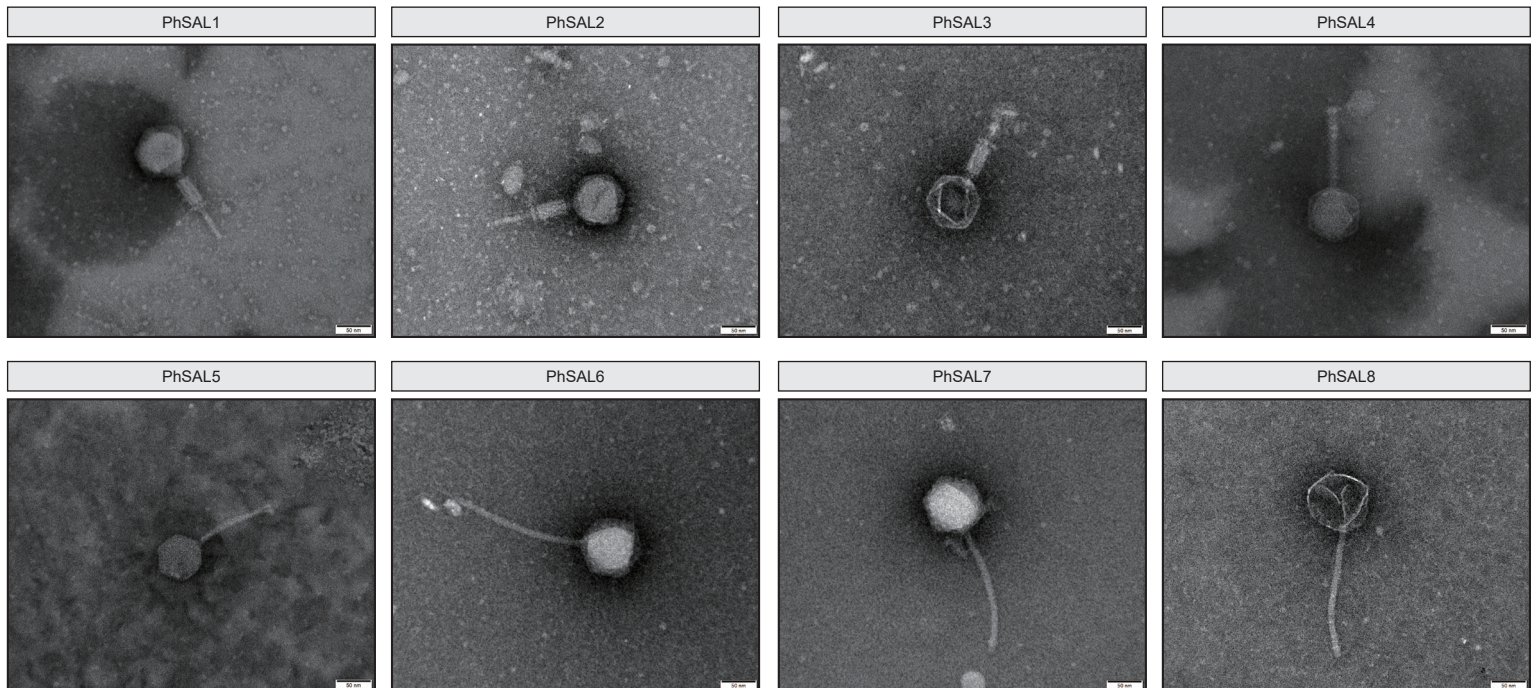

Supplement: Supplementary-Material_wrag077 [file supplementary-material_wrag077.zip › Fig_S1_wrag077.pdf]

**A**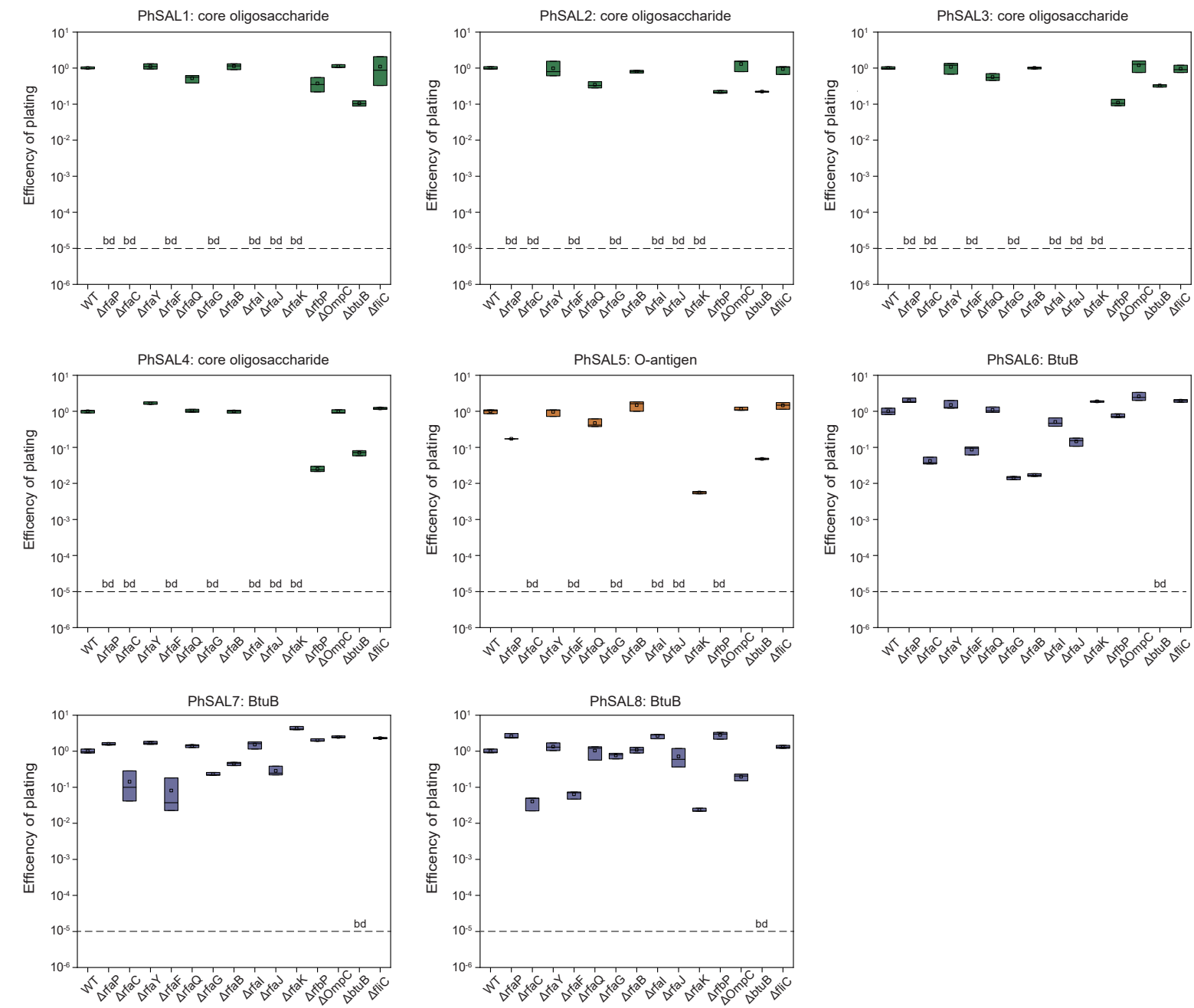**B**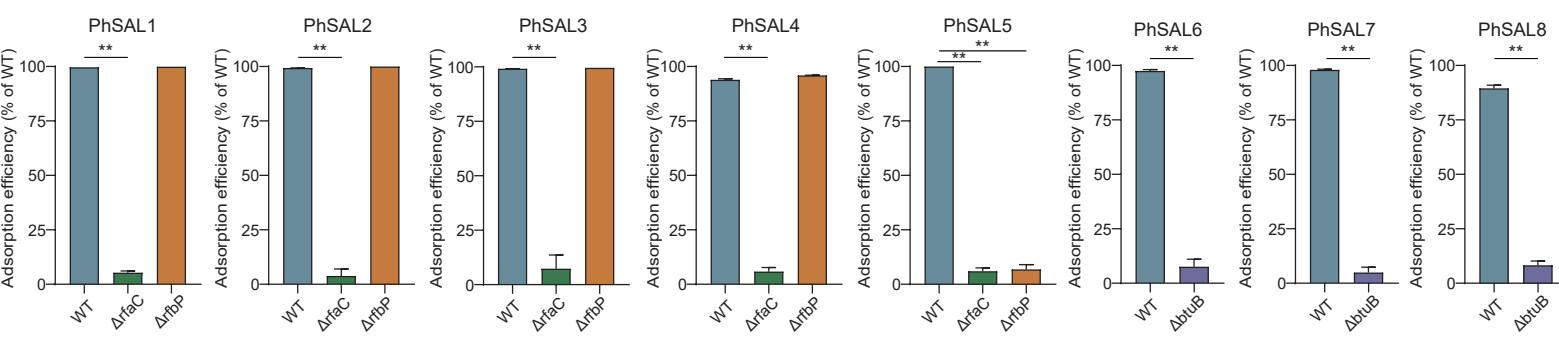

Supplement: Supplementary-Material_wrag077 [file supplementary-material_wrag077.zip › Fig_S2_wrag077.pdf]

# RBG Kernel Density Curve with Threshold and 95% CI

sensitive ( $\leq 0.49$ ), intermediate ( $0.49-0.53$ ), and resistant ( $\geq 0.53$ )

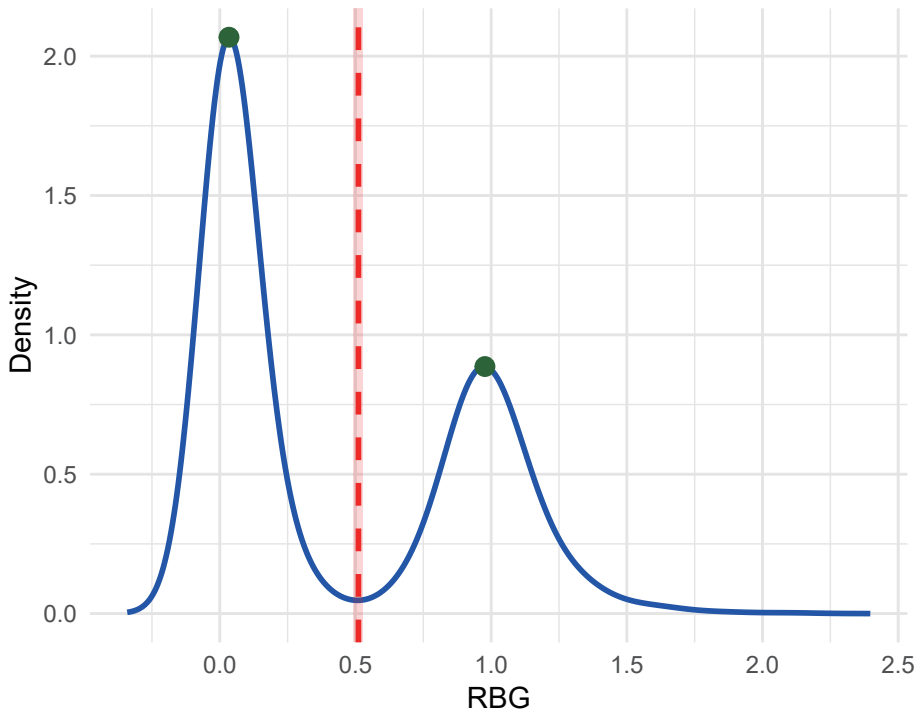

Supplement: Supplementary-Material_wrag077 [file supplementary-material_wrag077.zip › Fig_S3_wrag077.pdf]
