# Supplementary material for: Phage receptor specificity drives cross-resistance patterns and governs fitness trade-offs during sequential resistance acquisition in Salmonella
Source: ISME J. 2026 Apr 11;20(1):wrag077. doi: 10.1093/ismejo/wrag077 (PMC13196588; doi:10.1093/ismejo/wrag077)
Supplement: Supplementary-Material_wrag077 [file supplementary-material_wrag077.zip › Table_S1_wrag077.doc]

**Supplementary Table 1. Primers used to construct gene deletion strains**

| **Primer Name** | **Sequence(5'-3')** |
| --- | --- |
| *rfaP*-5arm-F | AGCAAAGAGCAATGTCCAGT |
| *rfaP*-5arm-R | CGCTGGCGTGACAAAGATT |
| *rfaP*-3arm-F | CAATTAAATCTTTGTCACGCCAGCGAGCTCTCTGAACACTTCGCC |
| *rfaP*-3arm-R | TGGAAGCCATTACTGCTGGT |
| *rfaC*-5arm-F | TTCCTGCAGCCCGGGGGATCAAAGCCATCGTCACCAACGA |
| *rfaC*-5arm-R | TTCCAAATACGCTTGTGGCACCCTTCTTCCGACAACCCAGT |
| *rfaC*-3arm-F | TGCCACAAGCGTATTTGGAAAG |
| *rfaC*-3arm-R | GAGTCAGTGAGCGAGGAAGCAAGAAACCCTCCAGTACCGT |
| *rfaY*-5arm-F | TTGAACAGAACCATTGACTGCAA |
| *rfaY*-5arm-R | AATTTCTTGCGTCGTATTAAGGGT |
| *rfaY*-3arm-F | TACCCTTAATACGACGCAAGAAATTAGCGCCGTAAACCCTTCTC |
| *rfaY*-3arm-R | CGCCCTGGAAAGATTCTCCT |
| *rfaF*-5arm-F | GCGCGACTTCGTTTACGTG |
| *rfaF*-5arm-R | GTGCGATAGAGACTTTGCGACA |
| *rfaF*-3arm-F | TGATGTCGCAAAGTCTCTATCGCACTTCGCTGTTGTCGGAAGAGG |
| *rfaF*-3arm-R | CTCCAGTCCATACCGTGCTT |
| *rfaQ*-5arm-F | CCTGATGACCTCGCGCC |
| *rfaQ*-5arm-R | TGGACCGACGACATCATTCAG |
| *rfaQ*-3arm-F | AAAACTGAATGATGTCGTCGGTCCATGCTGATGACAGGGGTGGTA |
| *rfaQ*-3arm-R | AGGCGCTGAACCCCATTATT |
| *rfaG*-5arm-F | TGATGTTCTGGTTAGCGGGT |
| *rfaG*-5arm-R | TACAGCTTACCGGAGAAGGC |
| *rfaG*-3arm-F | CCGCGGCCTTCTCCGGTAAGCTGTATGCAGACCGCCAAAAGGAAA |
| *rfaG*-3arm-R | ACTGGTCTGGCGGGTAAAAC |
| *rfaB*-5arm-F | TTAGCGAGGAGAACCGATGC |
| *rfaB*-5arm-R | ATTCCCGGCACGATTGAGAG |
| *rfaB*-3arm-F | AAAATCTCTCAATCGTGCCGGGAATATCTCTTTTAGCCATGCTTTATCCA |
| *rfaB*-3arm-R | ACTGGACATTGCTCTTTGCTTT |
| *rfaI*-5arm-F | AATGTGAAAAGCGAGCGGGA |
| *rfaI*-5arm-R | AGGCTAAGAGCAACTCACCG |
| *rfaI*-3arm-F | GACCACGGTGAGTTGCTCTTAGCCTAGAGACACCACAGCCAAAAAGA |
| *rfaI*-3arm-R | TGATTGTATGTCTGGCCCGC |
| *rfaJ*-5arm-F | CATAATAGAGATTTAGGCAGGGGAA |
| *rfaJ*-5arm-R | CATTATACGGGCGCTACAAAACC |
| *rfaJ*-3arm-F | ATGGTTTTGTAGCGCCCGTATAATGAGCGATTGATACCCCCACAC |
| *rfaJ*-3arm-R | ATATCGGACCAACGAAGCCC |
| *rfaK*-5arm-F | TCATCAGGGAAACAGCCAGC |
| *rfaK*-5arm-R | GCCACCAGATTGCCGAAAAA |
| *rfaK*-3arm-F | TTTGCTTTTTCGGCAATCTGGTGGCCCAGGTTTCTACCGCAGCC |
| *rfaK*-3arm-R | TTGCCCACGTTTCTATGACGA |
| *rfbP*-5arm-F | TTCCTGCAGCCCGGGGGATCTCCCAACTCGTTACACCCATTC |
| *rfbP*-5arm-R | CATCTCGCCGCAAAACAACCTAATCCTCACCCTCTGACCGT |
| *rfbP*-3arm-F | AGTTGTTTTGCGGCGAGATG |
| *rfbP*-3arm-R | GAGTCAGTGAGCGAGGAAGCGGATACGACGAGGCGTTTCGA |
| *OmpC*-5arm-F | TTCCTGCAGCCCGGGGGATCGTAGCGCCGAGGTCACGATA |
| *OmpC*-5arm-R | CGGCAACAAATTAGACCTGTGGCATCAACACCGACGACAT |
| *OmpC*-3arm-F | ACAGGTCTAATTTGTTGCCGTCT |
| *OmpC*-3arm-R | GAGTCAGTGAGCGAGGAAGCGCGGTCACTATTTTAGTTGCGAATG |
| *btuB*-5arm-F | TTCCTGCAGCCCGGGGGATCGCTGCGCCTCATACTGTTCT |
| *btuB*-5arm-R | CAAGCGGAAAAGGCCGTGA |
| *btuB*-3arm-F | GTCACGGCCTTTTCCGCTTGACGGTTGCATATCCGGTCAC |
| *btuB*-3arm-R | GAGTCAGTGAGCGAGGAAGCTAAGGACGTTTGACCGTGGC |
| *fliC*-5arm-F | TTCCTGCAGCCCGGGGGATCCGTCCTTTTTCAGCGTTGGT |
| *fliC*-5arm-R | AAGAGAGGACGTTTTGCGGATATTCTGGGTCAACAGCGACA |
| *fliC*-3arm-F | TCCGCAAAACGTCCTCTCTT |
| *fliC*-3arm-R | GAGTCAGTGAGCGAGGAAGCGCGGAAACGTAGTGCAGGT |
| *pabC*-5arm-F | ATCCCTTCCACAAACCGCAA |
| *pabC*-5arm-R | CAGGCAGGCGGATGAGATTAT |
| *pabC*-3arm-F | AATGATAATCTCATCCGCCTGCCTGGCGGTCGTAAAACTGCCATC |
| *pabC*-3arm-R | GCAGGCGGTTAAGTCAGTCT |
| *selB*-5arm-F | GGCGGAGGAATAAGCGAACA |
| *selB*-5arm-R | CCGGATTGGGTTTACACGAC |
| *selB*-3arm-F | ACGACGTCGTGTAAACCCAATCCGGCGGTAATCGCCTGGAGCAA |
| *selB*-3arm-R | GATTGCCCAACTGCAAAGCC |
| *tktA*-5arm-F | ACGCTTATCGGCAGAGGAAA |
| *tktA*-5arm-R | TTGTCGGGATGACCACCTTC |
| *tktA*-3arm-F | TCACCGAAGGTGGTCATCCCGACAATTTCTGTACTGCGTCCATGCT |
| *tktA*-3arm-R | GCTCAACGCTTTTACCTGCG |
| *wcaD*-5arm-F | CATTCTTTCCACCACCACAAC |
| *wcaD*-5arm-R | GGATAGTGGTGCGTTAGGTGA |
| *wcaD*-3arm-F | ACCTTCACCTAACGCACCACTATCCTGTGCCATTACTGCTTCCTGTT |
| *wcaD*-3arm-R | GCATCCAGCTTTACTGGGCT |
